# An experimental study on the intervention effects of group relay swimming training on social interaction deficits in adolescents with autism

**DOI:** 10.3389/fpsyg.2026.1976678

**Published:** 2026-10-13

**Authors:** YuFei Du, Yi Sun

**Affiliations:** College of Physical Education, Jilin University, Changchun, China

**Keywords:** adolescents with autism, ARSCW model, group relay swimming, interpersonal motor synchrony, peer rapport

## Abstract

Interventions addressing physical functioning and social generalization together remain scarce for adolescents with autism. This study developed the Aquatic Relay Social Coupling Window (ARSCW) model, which treats the instant of relay handoff as the smallest tolerable unit of social dosage and integrates sensory filtering by the aquatic medium, mandatory temporal coupling imposed by the relay format, and emotionally neutral collaboration driven by competitive goals. A 2 (training mode: group relay vs. traditional group swimming) × 3 (pairing strategy: two participants with autism, participants with autism paired with typically developing peers, and mixed teams) factorial randomized controlled design was adopted. A total of 224 adolescents with autism aged 12 to 17 years were randomized, 192 to the six factorial cells at 32 per cell and 32 to a wait list control group outside the factorial structure; 64 typically developing adolescents served as training partners only and provided no outcome data. Intervention and follow up each lasted 12 weeks, with a T2 retention rate of 88.4%. Underwater multi-person pose estimation yielded the Kuramoto order parameter and the window level index *W*_ARSCW_, its integral over the 15 s coupling window, expressed in seconds and computed under both training modes from structurally matched windows, combined with the Relay Handoff Social Coding system (RHSC), autonomic coupling and a self reported rating of peer rapport. Training mode showed a significant main effect and pairing strategy a significant moderating effect (*p* = 0.018); each relay cell separated from the wait list control group after correction while no traditional cell did. The increase in *W*_ARSCW_ mediated 51.7% of the total effect. Peer rapport followed the reverse ordering and was highest in two-autism pairings, so the objective and subjective dimensions dissociated. Social abilities generalized to physically embodied contexts, *d* from 0.39 to 0.68, whereas online effects were limited. The study provides empirical evidence and operational tools for adapted physical education curricula and team organization.

## 1 Introduction

Autism spectrum disorder (ASD) is a neurodevelopmental condition defined by difficulties in social communication and by restricted or repetitive behaviors, with prevalence among children aged 8 years now reported at 1 in 36 (Maenner et al., 2023). In adolescence, peer acceptance, body image and self concept change together, so isolation, anxiety and depression frequently co-occur (Bhat, 2021). Motor and social limitations are mutually entangled rather than parallel, which makes an intervention that improves physical functioning while naturally embedding social interaction an urgent practical need.

Physical exercise benefits core autism symptoms in meta-analyses of randomized trials (Wang et al., 2025), and the buoyancy, hydrostatic pressure and sensory filtering of water make aquatic exercise a common choice: technique or game centered programs improve gross motor skills and repetitive behaviors (Marzouki et al., 2022) and Halliwick based trials report gains in aquatic skill and balance (Vodakova et al., 2022). Such work has predominantly used one-to-one coaching with younger children and limited its goals to water safety, so social outcomes remain inconsistent (Kemp et al., 2024). Land based group exercise offers an alternative, since mini-basketball training improves social communication alongside changes in brain function and white matter integrity (Yang et al., 2024; Cai et al., 2020) and group play demonstrates the independent value of peer contexts (Xing et al., 2025). Interpersonal motor synchrony is generally impaired in autism, measurable from the preschool years (Chen et al., 2023) and underlying social interaction difficulty (Carnevali et al., 2024). Group relay swimming combines rhythmicity, task orientation and competition in a form of aquatic peer coordination whose effects and mechanisms remain unexplored.

This study proposes the ARSCW model, which identifies the relay handoff as the smallest tolerable unit of social exposure and integrates three mechanisms: sensory filtering reduces the burden of eye contact and facial recognition, mandatory temporal coupling creates controllable bursts of interaction, and emotionally neutral cooperative motivation promotes active coordination. A 2 × 3 factorial randomized controlled design elevated relay pairing composition to an independent design factor. One hundred and ninety two adolescents with autism entered the six cells, 32 formed a wait list control group outside the factorial structure, and 64 typically developing adolescents served as training partners only. Methodologically the study developed a multi-person synchrony algorithm from underwater multicamera pose estimation, constructed the RHSC and introduced autonomic coupling measurement with waterproof wearables. Self reported peer rapport was recorded alongside these objective channels, so that the consequences of team composition could be traced on an objective and a subjective dimension at once.

## 2 Literature review

### 2.1 Paradigm bottleneck: structural disconnection between individualized instruction and social generalization in aquatic exercise interventions

Systematic reviews of aquatic interventions reveal an imbalance across populations, outcomes and delivery modes. Most programs follow a one-to-one coaching paradigm with same-age peers almost absent (Failla et al., 2026) and prioritize water safety over social outcomes. Holistic movement therapies add rhythm and music and yield small to moderate social communication effects (Amonkar et al., 2021), but their benefit derives from unidirectional therapist-to-child guidance and lacks a reproducible unit of repeated peer exchange. Peer-mediated interventions are effective in classrooms and playgrounds (Zhang et al., 2022) yet absent from aquatic settings, and dyadic exercise interventions such as yoga confine the unit to a therapist-child pair (Kaur et al., 2021). Adolescent samples are scarce, since most studies end at the preschool stage (Salar, 2024).

### 2.2 Mechanism bottleneck: existing theories do not explain the unique pathway of mandatory social moments in relay events

Deficits in interpersonal motor synchrony (IMS) in autism are characterized by weak coupling strength and poor phase alignment and are associated with social competence (McNaughton and Redcay, 2020). Most experiments rely on laboratory tasks such as pendulum swinging and finger tapping that are detached from real sport (Glass and Yuill, 2024b). Dyadic joint action work shows that synchrony is associated with peer social initiation but is strongly moderated by task type (Estrugo et al., 2023), and synchronized drumming with functional near-infrared spectroscopy shows that creative movement enhances social activation in the inferior frontal gyrus and superior temporal sulcus (Su et al., 2025). Although reviews note the effect of sport on social and motor skills (Levante et al., 2023), no model addresses the temporal structure formed by competitive rhythm, mandatory handoff and immediate separation, which relay swimming compresses into periodic momentary events.

### 2.3 Measurement bottleneck: persistent lack of objective quantification of multi-person motor synchrony in aquatic environments

Social outcomes have long been measured with parent-completed scales such as the SRS-2, whose results are influenced by caregiver subjectivity and adolescent camouflaging. Computer vision has produced underwater pose estimation for individual swimmers with subcentimeter accuracy (Giulietti et al., 2023), but almost all systems process a single swimmer and none estimates relative phase when several share the frame under occlusion and splash. Wearable sensing can capture stress and emotional fluctuation through electrodermal activity and heart rate variability (Cano et al., 2024), movement variability predicts developmental trajectories (Wilson et al., 2024), and observing synchrony recruits the action observation network in a manner not wholly impaired in autism (Bierlich et al., 2024). These tools have never been integrated for multi-person social coupling in water.

### 2.4 Population and team formation bottleneck: scarcity of adolescent samples and lack of systematic testing of team composition strategies

Peer combination influences social synchrony and rapport. Self-ratings of rapport in two-autistic pairings are not inferior to those in two-typically-developing pairings, whereas cross-neurotype interactions may involve the greatest friction (Crompton et al., 2020), and non-autistic observers display systematic bias when evaluating such interactions (Jones et al., 2024). These findings concern how an interaction feels from inside it, whereas the kinematic literature concerns how tightly movements align in time; the two need not move together, because alignment rewards complementarity in motor skill while felt rapport rewards shared communicative style. Peer type and activity type jointly determine social motor synchrony (Glass and Yuill, 2024a), and adolescent camouflaging causes scale scores to diverge from subjective experience (Lei et al., 2024). Reviews report sample shortages, uneven methodological quality and a lack of transfer tests (Shahane et al., 2024), while anxiety and depression rise linearly with age (Uljarević et al., 2020). Existing group exercise interventions have uniformly used random grouping. The objective and subjective dimensions have moreover almost never been measured within a single trial, so the possibility that one composition is superior on one and inferior on the other has remained untested.

### 2.5 Research hypotheses and theoretical model

These limitations converge on four questions: whether group relay swimming works as a standalone intervention, through which mechanism, under which boundary conditions, and whether the acquired abilities transfer. Four testable hypotheses were proposed:

**H1:** Compared with traditional group swimming, group relay swimming will significantly improve the core social interaction outcomes of adolescents with autism, with concordant changes in SRS-2 scores and structured behavioral observation indicators.
**H2:** The intervention effect will be mediated by improvement in underwater multi-person motor synchrony, and IMS indicators will significantly mediate the relationship between training mode and social outcomes.
**H3:** Relay pairing strategy will moderate the intervention effect. Mixed teams and pairings between participants with autism and typically developing participants will produce greater gains in objective synchrony indicators than pairings between two participants with autism, whereas, self reported peer rapport will follow the reverse ordering and will be highest in pairings between two participants with autism, so that the two dimensions dissociate rather than rise together.
**H4:** Social abilities acquired through the intervention will transfer to non-aquatic contexts, and the degree of transfer will be jointly predicted by baseline synchrony and gains in synchrony during training.

The ARSCW model is presented in Figure 1. The horizontal axis presents preparation, handoff and departure; the vertical axis lists the input channels of sensory filtering, mandatory temporal coupling and competitively driven collaboration. These converge at the coupling window node, which incorporates four measurement channels comprising kinematic synchrony, behavioral coding, autonomic coupling and self reported peer rapport, connected by solid arrows to social interaction, physical performance and mental health. Pairing strategy is a dashed moderating path affecting the width and depth of the window, splitting into one branch to the objective channels and one to the rapport channel with opposite signs to express the dissociation predicted in H3. Arrows labeled H1 to H4 map each hypothesis to its pathway.

**Figure 1:**
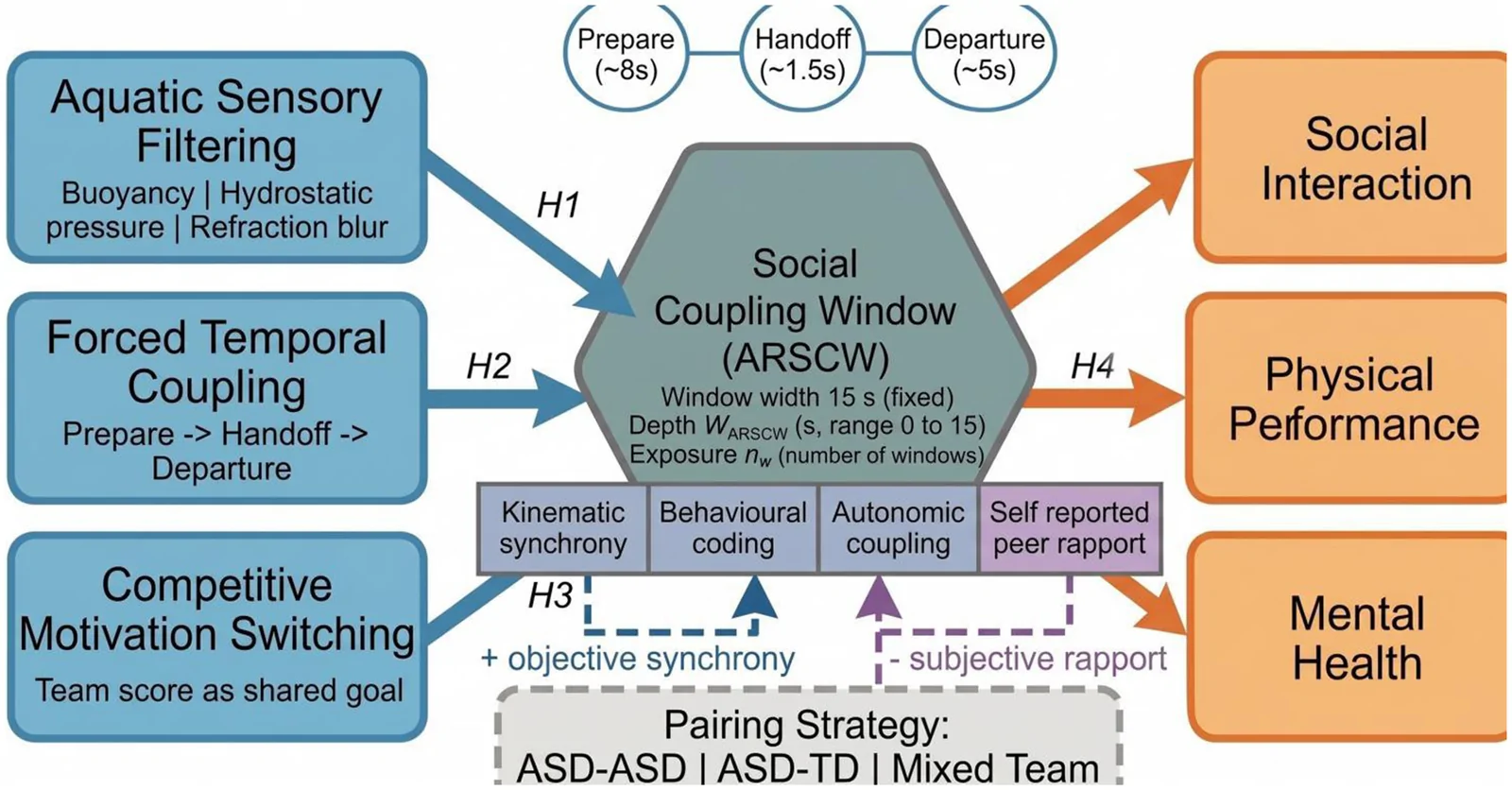
The Aquatic Relay Social Coupling Window (ARSCW) theoretical model.

## 3 Research design and methods

### 3.1 Operationalization of the aquatic relay social coupling window model

The first mechanism, sensory filtering, operates through the physical properties of water: buoyancy relieves the postural-control burden, hydrostatic pressure delivers uniform proprioceptive input, and refraction softens facial detail and eye-contact cues that many adolescents with autism find aversive. Peer-coordination tasks were conducted in 1.2 to 1.4 m lanes at 28 to 30 degrees Celsius under dimmed lighting.

The second mechanism, mandatory temporal coupling, is imposed by the relay format. Each handoff unfolds as a three-beat cycle of preparation (~8 s), handoff (~1.5 s) at wall contact and departure (~5 s), forming a ~15-second coupling window treated as the smallest tolerable unit of social dosage, whose cumulative number across the 12-week intervention, denoted *n*_*w*_, is the key exposure indicator. The same structure has a counterpart in traditional group swimming, where a swimmer approaching the wall and a teammate in the same lane occupy a window of the same length at the same lateral separation, so both arms share one analytical unit.

The third mechanism, motivational switching, embeds the social demand in a competitive team goal, so that synchronizing with a peer becomes a practical condition for team success rather than a socially loaded expectation; coaches used performance cues such as “match the rhythm” rather than social cues such as “make eye contact.” The depth of synchrony sustained inside a window and the number of windows delivered per session are recorded as two separate quantities and together describe the social dose, allowing the arms to be compared on structured social exposure rather than total pool time.

### 3.2 Participants and ethical procedures

Adolescents with autism aged 12 to 17 years were recruited from special education schools, autism support organizations and adolescent psychiatric outpatient clinics in three neighboring cities, through information sessions followed by an eligibility screening interview with a research nurse trained in autism-friendly communication.

Inclusion criteria were age 12 to 17 years, a DSM-5 diagnosis of autism spectrum disorder confirmed jointly by ADOS-2 and ADI-R, full-scale intelligence quotient of 70 or above, basic water competence, and written caregiver consent with adolescent assent. Exclusion criteria were structured swimming training within the previous 6 months, uncontrolled epilepsy, moderate to severe cardiovascular disease, severe tactile or auditory hypersensitivity, and concurrent enrollment in another structured social skills program.

For the two pairing conditions that included typically developing peers, 64 adolescents in the same age range were recruited from local secondary schools, screened for the absence of developmental, psychiatric or chronic physical conditions, and briefed on the collaborative nature of the program. Thirty two peers were assigned to the group relay arm and 32 to the traditional arm, and no peer moved between arms. Within an arm each peer served in exactly two four person teams, one in the autism to typical development condition and one in the mixed team condition, and never twice within the same condition. Peer identity was therefore reused across teams by design and was logged for every team place, so that the resulting dependence could be modeled explicitly. Peers attended training sessions only and were excluded from the main analysis.

The protocol was approved by the Institutional Review Board of the coordinating university and by the ethics committees of each participating school. Retention was supported by weekly schedule messages, transport vouchers for distant families and same-day contact with a make-up slot whenever a session was missed.

### 3.3 The group relay swimming intervention protocol

The intervention lasted 12 weeks with three 90-min sessions per week (36 sessions). Each session followed a fixed sequence: 10 min dry-land warm-up and preview on a visual schedule board, 15 min individual technique work, 45 to 50 min stage-specific coordination or relay content, 10 min poolside debriefing and 5 to 10 min cool-down. The number of handoffs, and therefore of coupling windows, rose progressively across four stages from zero to approximately 387 cumulative windows.

The four stages progress from aquatic adaptation in weeks 1 to 3, with individual techniques side by side and no handoff, through dyadic coordination in weeks 4 to 6, with metronome-guided synchronized strokes and about eight paired coordination moments per session, to small-group relay in weeks 7 to 9, with 25-meter shuttle relays and a 30-second review after each handoff, and full-team competition in weeks 10 to 12, with 4 by 50-meter race simulation in which the coach acts only as adjudicator and safety officer. Stage-specific content, coaching scripts and scaffolding withdrawal are detailed in Supplementary material 1.

As shown in Figure 2, per-session handoffs rose from zero in stage I to approximately 24 in stage IV while coach intervention density declined in the opposite direction, a joint pattern of rising participant-driven exposure and declining adult scaffolding that distinguishes the ARSCW program from conventional aquatic interventions.

**Figure 2:**
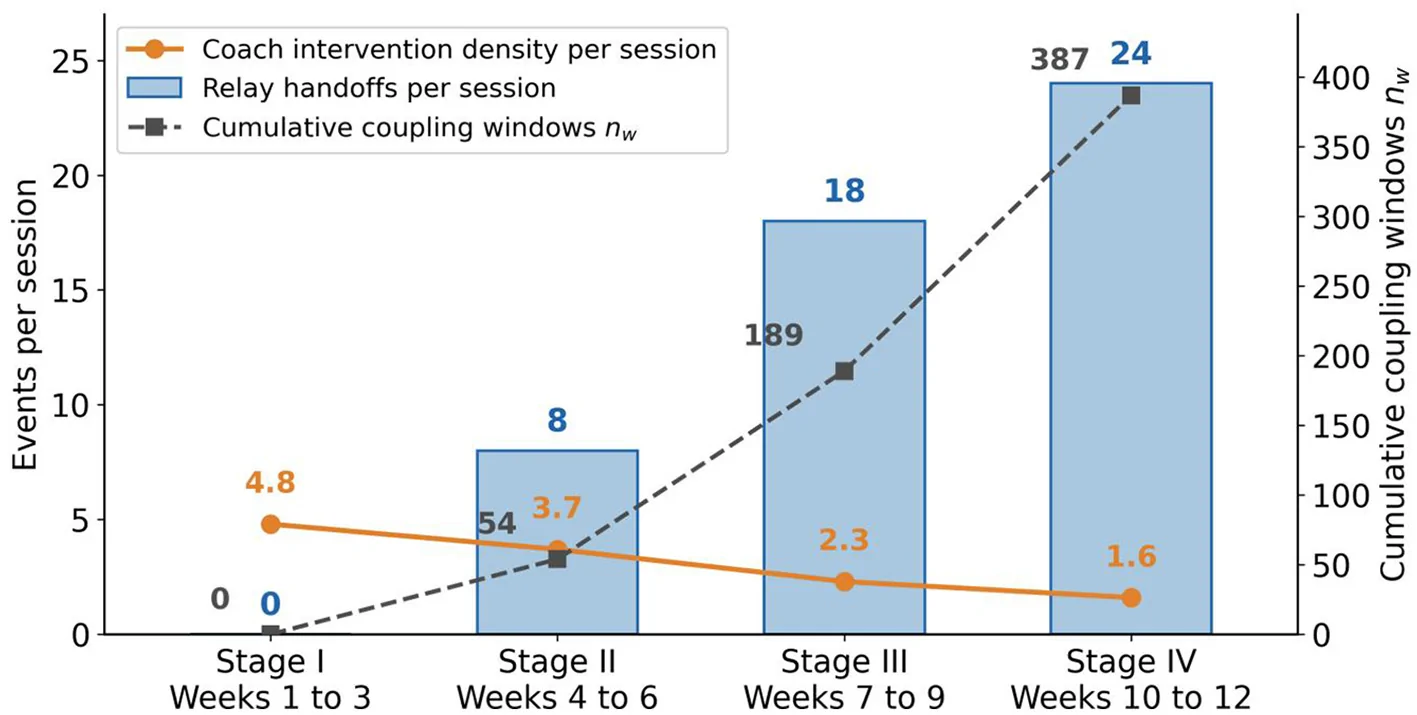
Staged structure and cumulative coupling windows of the group relay swimming intervention across 12 weeks.

Table 1 summarizes the training content and dosage parameters for each stage. The traditional group swimming condition served as the active comparator, matched on total pool time, coach-to-participant ratio, lane assignment and technique curriculum, and followed a standard lesson format of parallel technique drills, individual endurance sets and free-swimming intervals.

**Table 1:** Staged training content and coupling window dosage of the group relay swimming intervention.

Stage	Weeks	Core training content	Relay handoffs per session	Coach intervention density	Cumulative coupling windows *n*_*w*_
I Aquatic adaptation	1 to 3	Individual techniques, side by side swimming, and water familiarity	0	High (demonstration and verbal commands)	0
II Dyadic coordination	4 to 6	Synchronized strokes guided by a metronome, mirrored kicking, and *R* value feedback	8	Moderate (rhythmic guidance)	Approximately 54
III Relay in small groups	7 to 9	Relay with three swimmers, handoff at wall contact, and verbal review	18	Moderately low (review facilitation)	Approximately 189
IV Competition with full teams	10 to 12	4 × 50 m race simulation, interteam competition, and points system	24	Low (race adjudication)	Approximately 387

All coaches were licensed instructors with at least 3 years of experience teaching adolescents and completed a 30-h preparatory program covering the ARSCW rationale, autism-friendly communication, visual schedule use and the stage-specific script. Fidelity was assessed by a signed script-review checklist before every session, an independent evaluator attending a random 20% of sessions and rating adherence on a 0-to-10 scale, and a masked assessor reviewing a random 10% of archived videos.

Values in the final column are cumulative counts of the exposure variable *n*_*w*_ for one participant, expressed in windows, and are distinct from the depth index *W*_ARSCW_, expressed in seconds. Handoffs per session are the value reached at the end of each stage and rise progressively within it, so the cumulative column accumulates the session means across the nine sessions of each stage.

### 3.4 The 2 × 3 factorial randomized controlled design

The study adopted a 2 (training mode: group relay vs. traditional group swimming) × 3 (pairing strategy: two participants with autism, participants with autism paired with typically developing participants, and mixed teams) factorial randomized controlled design, giving six intervention cells. An additional Wait-list Control (WLC) group, which received the relay program after study completion, estimated natural change and offered an ethical option for interested families. The central design innovation was to elevate relay pairing composition to an independent factor combined orthogonally with training mode.

Each pairing condition was defined by the composition of the four person team and by the neurotype of the partner on each side of a handoff. In the two-autism condition all four members were adolescents with autism, so every handoff joined two participants with autism. In the autism to typical development condition a team held two adolescents with autism and two peers in a strictly alternating order, each adolescent with autism keeping one fixed peer partner in the adjacent position for all 36 sessions, so every handoff crossed neurotype with an unchanging partner. In the mixed team condition a team also held two adolescents with autism and two peers, but the two adolescents with autism were adjacent and the order was rotated weekly, so each met both a same neurotype and a cross neurotype partner and held no fixed partner. The second and third conditions therefore differ in partner stability and in the neurotype mixture of the handoffs, not in the number of peers present. Table 2 gives composition, allocation and clustering structure: across both arms the design comprised 80 teams and 192 adolescents with autism, the 64 peers filled 128 team places, and the 32 of the WLC group were not allocated to any team.

**Table 2:** Team composition, typically developing peer allocation and clustering structure of the three pairing conditions.

Pairing condition	Composition of one team	Neurotype of handoff partner	Partner assignment	Teams per training mode arm	Participants with autism per cell	Peer places per training mode arm
Two participants with autism	4 participants with autism	Same neurotype at every handoff	Fixed running order within the team	8	32	0
Participants with autism and typical development	2 with autism and 2 peers, alternating order	Cross neurotype at every handoff	One fixed peer partner for 36 sessions	16	32	32
Mixed team	2 with autism and 2 peers, the two adjacent	Same and cross neurotype within one team	Order rotated weekly, no fixed partner	16	32	32
Wait-list control	No team allocation	Not applicable	Not applicable	0	32	0

Sample size was estimated by a priori power analysis targeting the 2 × 3 interaction, which required the largest sample among the planned tests. The computation used G^*^Power 3.1, F test family, routine for main effects and interactions in fixed effects analysis of variance (Faul et al., 2007). With *f* = 0.25, corresponding to a partial η^2^ of 0.059, a two sided α of 0.05, a target power of 0.80, a numerator degree of freedom of 2 and six cells, the required total sample was 158, with noncentrality parameter λ = *f*^2^*N* = 9.88 and achieved power 0.802; 158 divided by six cells gives 26.3 per cell, rounded up to 27. The target was raised to 32 per cell for 15 percent anticipated attrition, giving 192 adolescents with autism in the intervention arm and 224 in total with the WLC group. The 64 peers were training partners only and not part of the randomized sample. Randomization used a central web-based platform managed by an independent statistician who took no part in recruitment, delivery or assessment. The fixed block size of 14 is two allocations for each of the seven groups formed by the six cells and the WLC group, so every completed block preserved exact balance across all seven groups; stratification by age band, sex and baseline SRS-2 tertile yielded 12 strata, each with an independent permuted block sequence, and a recruitment cap of 32 per group produced the final allocation of 32 per group. The full stratum and block implementation is given in Supplementary Material S2.

Because the relay team rather than the individual was the unit of delivery, and each peer served in two teams, dependence was anticipated at two levels. Team membership and peer identity were recorded for every team place, and all factorial models were specified in advance as linear mixed models carrying a random intercept for relay team and a crossed random effect for peer identity, alongside the fixed effects model of the same structure; the team level intraclass correlation and design effect are reported with the fixed effects.

Because the intervention was physical, participants and coaches could not be blinded. All outcome assessors, data-entry personnel and analysts were masked to allocation and inadvertent disclosures were logged as protocol deviations. Assessors received two-day training in autism-friendly assessment and had to reach at least 90% agreement with a criterion rater. Assessments were conducted at baseline (T0), mid-intervention (T1, end of Week 6), post-intervention (T2, end of Week 12) and follow-up (T3, 12 weeks later), with T3 adding non-aquatic context tasks for Hypothesis 4. Figure 3 presents the CONSORT-style flow, in which the 192 adolescents with autism of the six cells, the 32 of the WLC group and the 64 peers appear as three separate streams, and every allocation box carries both the randomized sample entering the intention to treat analysis and the sample observed at that time point.

**Figure 3:**
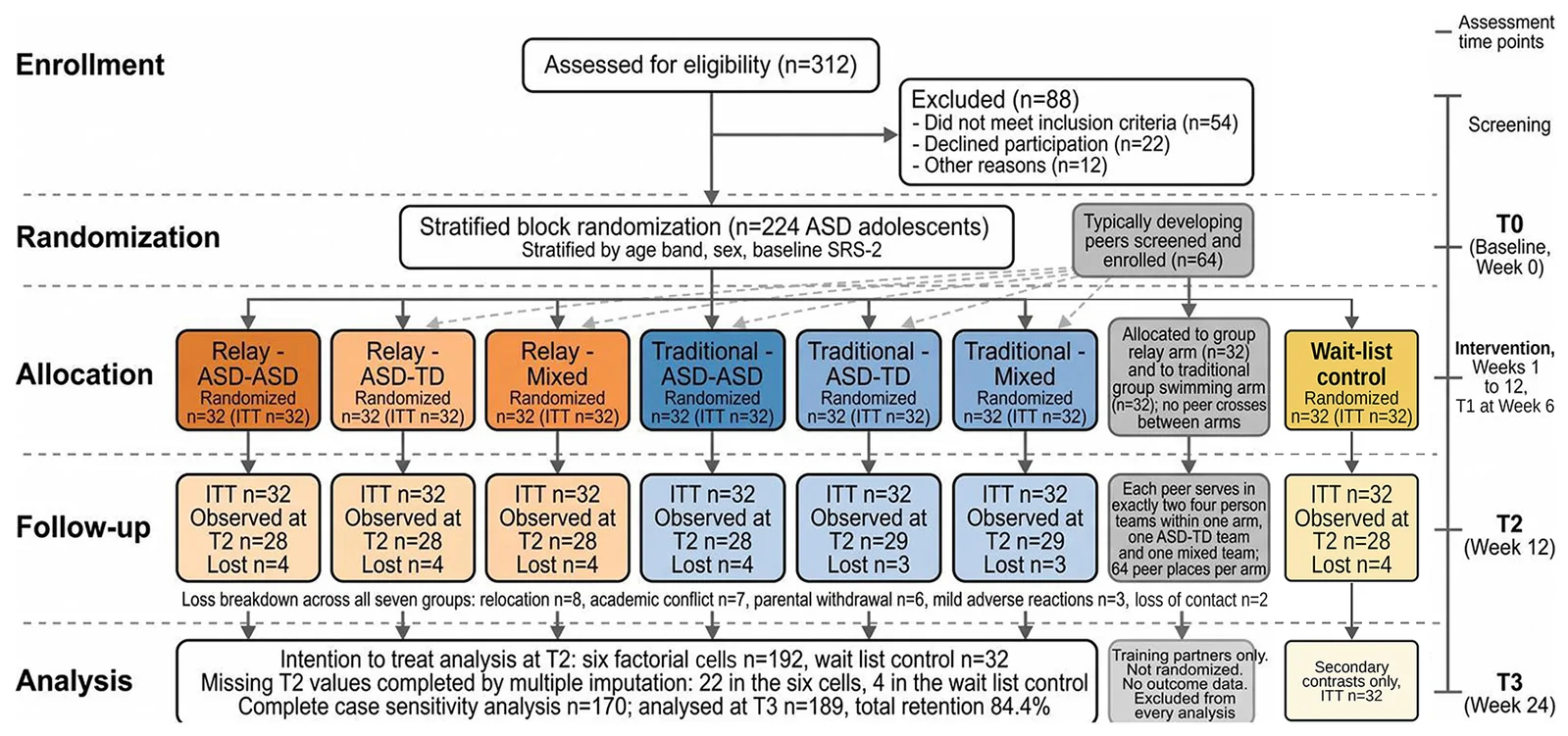
The 2 × 3 factorial randomized controlled design and CONSORT flow diagram.

### 3.5 Outcome measures and data collection procedures

Because caregiver-report scales are affected by camouflaging and rater fatigue, a multi-channel system combined a caregiver-report instrument, direct behavioral observation at the handoff, kinematic quantification of team synchrony, wearable physiological monitoring and a self reported rating of peer rapport. The five channels converged on the coupling window as the shared unit of analysis, and all streams were clock-aligned.

(1) Primary caregiver-report outcome: SRS-2

The SRS-2, a validated 65-item questionnaire capturing reciprocal social behavior across five subdomains, was the primary caregiver-report outcome, completed by the same caregiver at all four time points; its T0 to T2 change anchored effect-size interpretation.

(2) Direct behavioral coding: the Relay Handoff Social Coding system (RHSC)

The RHSC was developed for this study to capture social microbehaviors within the 15-second window surrounding each handoff, organizing 12 secondary codes under visual attention, body posture, nonverbal feedback and verbal exchange. Each window was divided into one-second intervals and two independent coders made a binary present-or-absent judgment for each code. For the traditional condition, structurally comparable 15-second segments around each swimmer's turn at the pool end were coded with the same system, so between-condition comparisons used matched observation windows. The same matched windows serve the kinematic and physiological channels below, so every index defined on the coupling window carries an observed value in both arms.

Coder reliability was assessed with Cohen's κ; only codes with κ ≥ 0.80 entered formal analysis and codes below threshold were re-adjudicated by a third coder. Figure 4 illustrates the hierarchical organization of the RHSC and the coding workflow.

(3) Kinematic synchrony: underwater multi-person motion capture

**Figure 4:**
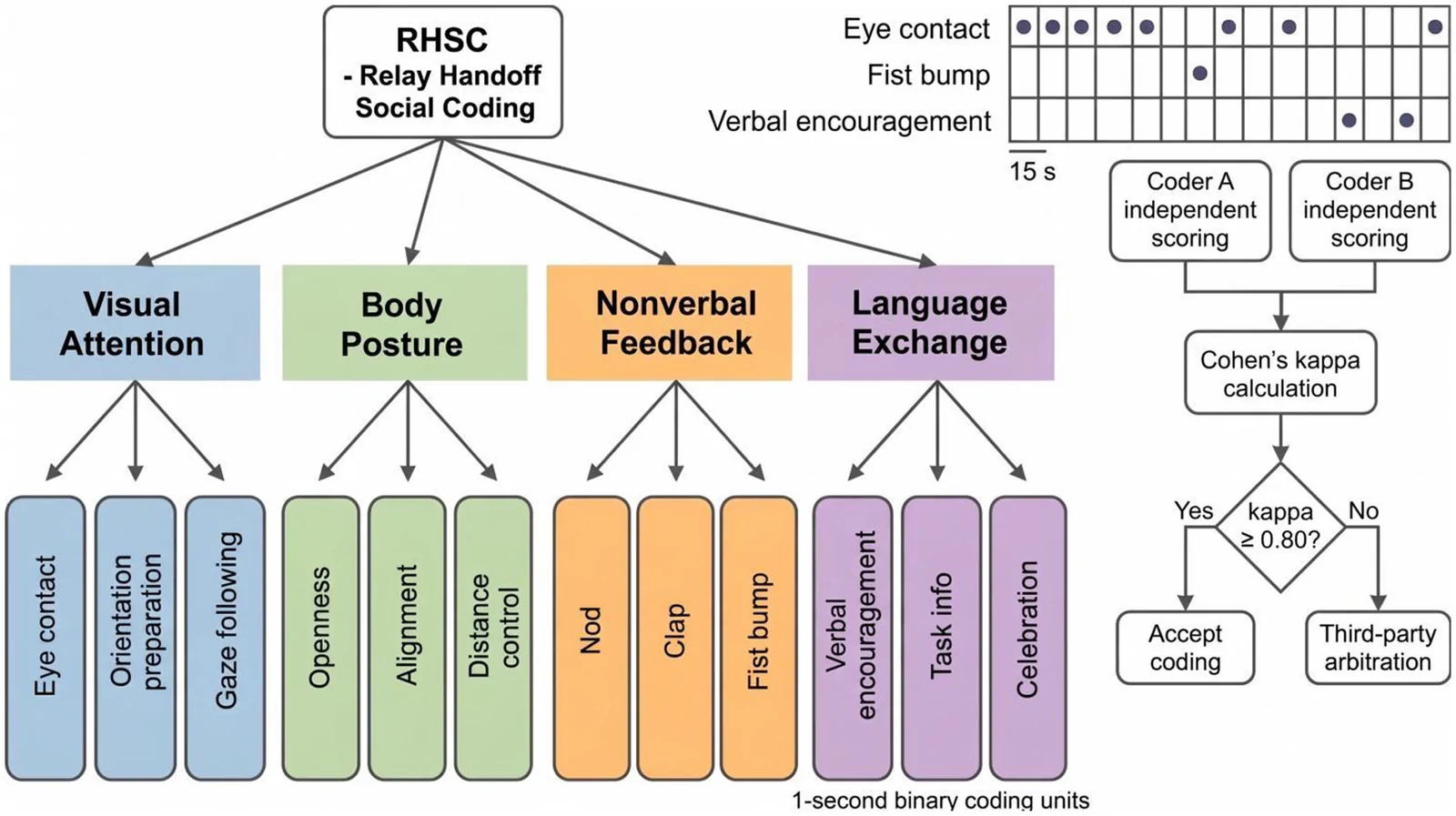
Structure of the RHSC relay handoff social coding system.

Kinematic synchrony was quantified with an underwater multi-camera system: two waterproof high-speed cameras below each side wall plus one overhead camera gave five viewpoints covering the water column and swimming plane, recording at 120 fps in 4K and calibrated at each session start. A validated pose estimation pipeline reconstructed each swimmer's three-dimensional trajectory, identity being maintained by combining appearance features with temporal trajectory continuity when swimmers overlap or splash occludes the frame.

From the reconstructed traces the Kuramoto order parameter *R*(*t*) was computed as the primary index of team synchrony. *R*(*t*) summarizes momentary phase alignment across the swimmers coupled at a given instant on a 0-to-1 scale and is defined as follows:


$$\begin{array}{l} R\left(t\right)=|\frac{1}{N}\overset{N}{\underset{k=1}{\sum }}e^{i\theta_{k}\left(t\right)}|\; \end{array}$$ (1)

In Equation (1), *N* is the number of swimmers inside the coupling window under analysis rather than the nominal team size. During a handoff only two members are temporally coupled, the swimmer arriving at the wall and the swimmer leaving the block, so *N* = 2 for every coupling window, and this is the value used for the window index and the mediation test. The team level value with *N* = 4 is defined only for the aligned start of the 4 × 50 m race simulation and is reported descriptively.

Phase was not read off the stroke cycle, because a swimmer waiting on the block performs no stroke. A continuous oscillatory kinematic signal was extracted instead: anteroposterior wrist displacement for a swimmer traveling in the water and vertical shoulder displacement for a swimmer on the block, which tracks the preparatory rocking cycle. Both were filtered with a fourth order zero phase Butterworth band pass between 0.3 and 2.5 Hz, and the instantaneous phase was taken from the analytic signal generated by the Hilbert transform, following the procedure established for multi person motor synchrony (Smykovskyi et al., 2022):


$$\begin{array}{l} \theta_{k}\left(t\right)=\arctan\frac{H\left[x_{k}\left(t\right)\right]}{x_{k}\left(t\right)} \end{array}$$ (2)

In Equation (2), *x*_*k*_(*t*) is the filtered kinematic signal of swimmer *k* and H[·] denotes the Hilbert transform. Phase reconstruction for the swimmer on the block was validated against frame by frame manual annotation on a random 10 percent of windows, giving a mean absolute phase error of 0.21 rad and a cycle detection rate of 94.6 percent; windows with fewer than 60 percent of required keypoints were discarded, retaining 88.9 percent.

R(t) served as real-time process feedback for coaches and participants at the end of each set and, through its integral over the coupling window, as the mediator for testing Hypothesis 2. The window level synchrony index is defined as


$$\begin{array}{l} W_{\text{ARSCW}}=\frac{1}{J}\overset{J}{\underset{j=1}{\sum }}\int_{t_{j}}^{t_{j}+\Delta }R\left(t\right)\text{d}t,\Delta =15\;s \end{array}$$ (3)

In Equation (3), *t*_*j*_ is the onset of the *j* th window, Δ the fixed window length of 15 s and *J* the number of windows entering the average. Because *R*(*t*) is bounded between 0 and 1, *W*_ARSCW_ carries the unit of seconds, is bounded between 0 and 15 s and equals 15 s multiplied by the mean of *R*(*t*) inside the window, so it expresses the depth of synchrony sustained within one window. The cumulative window count *n*_*w*_ is a separate exposure variable in units of windows and enters only the dose response analysis of Hypothesis 4, so depth and exposure are never merged. The integral was evaluated by trapezoidal summation over the 120 Hz samples and averaged over the windows of weeks 10 to 12. The baseline value came from the same pipeline applied to the matched turn windows of the T0 swimming assessment, in which each participant swam with the team assigned by randomization and no handoff was delivered.

Both arms yield an observed value of *W*_ARSCW_. In the relay condition the window is the handoff; in the traditional condition it is the matched segment at the pool end, where a swimmer approaching the wall and a teammate in the same lane share the same 15 s interval at the same lateral separation, so Equation (1) is applied to the same number of swimmers over a window of the same length. The pair inside a matched turn window is defined by team structure rather than proximity: it joins the participant with the teammate occupying the adjacent position in the same team, the pair that would exchange a handoff if that team were running a relay, so the pairing strategy factor retains its meaning in both arms. In weeks 10 to 12 the traditional condition yielded 22 to 26 matched windows per session against 24 relay handoffs, and the same segments already serve as the observation unit of the behavioral coding, which returns non zero counts there. Every value in both arms was computed from recorded kinematic data through an identical pipeline, the arms differing only in what opens the window. Because the window is the moment of closest alignment, the mean of *R*(*t*) inside it exceeds the session mean, and the two are reported separately. Figure 5 shows the signal chain and the correspondence between a handoff window and a matched turn window.

(4) Physiological coupling: wearable monitoring

**Figure 5:**
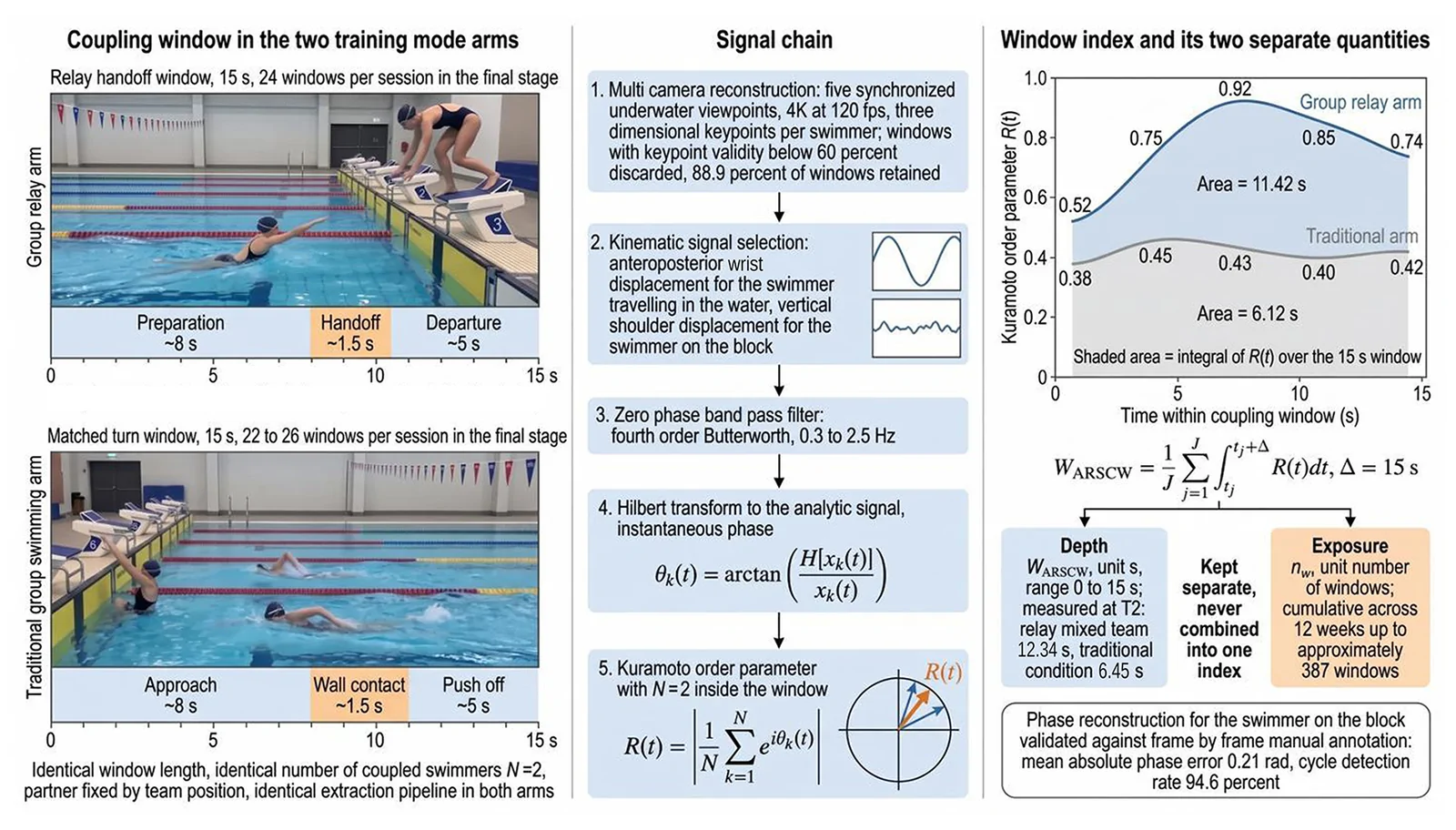
Phase extraction and computation of the coupling window synchrony index in the two training mode arms.

Autonomic coupling was measured with Polar H10 chest straps for heart rate and heart rate variability and Empatica EmbracePlus wristbands for electrodermal activity. The strap was worn beneath the swimsuit and stored RR intervals at 1000 Hz, a configuration whose agreement with electrocardiography is established at rest and across graded exercise (Schaffarczyk et al., 2022) and whose signal quality in the pool has been examined against wrist worn alternatives (Cosoli et al., 2022). Because a submerged electrode pair cannot separate sweat gland conductance from that of the surrounding water, the electrodermal analysis was restricted to samples with the wrist above the water surface, identified from triaxial acceleration and the wet contact flag, so that it describes the dry phases of the window.

RR series were corrected for ectopic and missed beats at a 0.25 s threshold and windows requiring correction of more than 5 percent of beats were discarded, retaining 86.2 percent on the cardiac channel. Electrodermal signals were low pass filtered at 1 Hz and motion artifacts labeled by a supervised detector using signal features and triaxial acceleration (Hossain et al., 2022); windows with more than 10 percent artifact samples were discarded, retaining 79.4 percent, of which 61.8 percent of all windows also met the above water criterion.

The cardiac quantity entering the coupling analysis was the instantaneous heart rate series resampled at 4 Hz, not a spectral index, which is undefined over 15 s; at the observed window heart rates of 140 to 170 beats per minute a 15 s segment contains 35 to 43 RR intervals, comparable to a 30 s resting segment, so the root mean square of successive differences computed over the window agreed with a 5 min reference from the same session at *r* =0.84, consistent with evidence that time domain variability is recoverable from ultra short recordings during exercise (Tanoue et al., 2023). For each pair of teammates, coupling strength was the peak of a time-lagged cross-correlation within the same 15-second window, with lags up to three seconds in either direction, that is


$$\begin{array}{l} C=\underset{|\tau |\leq 3s}{\max}\rho \left[x_{p}\left(t\right),x_{q}\left(t+\tau \right)\right] \end{array}$$ (4)

In Equation 4, *x*_*p*_ and *x*_*q*_ are the standardized signals of the two teammates and ρ the Pearson correlation over the window. Devices were fitted before each session, participants had a five-minute quiet-standing baseline, and all signals were clock-aligned within 10 milliseconds. Observed coupling exceeded the 95th percentile of 1000 phase randomized surrogates in 81.7 percent of cardiac and 68.3 percent of electrodermal windows, and a repeated measurement substudy with 24 participants across three sessions returned ICC(2,1) of 0.74 and 0.66. Because the electrodermal channel retained fewer windows, described only the dry phases and was less repeatable, cardiac coupling was treated as the primary physiological index and electrodermal coupling as secondary and exploratory. Complete preprocessing, artifact rejection and reliability statistics are reported in Supplementary material 3.

(5) Transfer assessments at T3

To evaluate transfer, five assessments were added at T3: a peer cooperation task at school recess, naturalistic observation at a family gathering, free-activity observation at a community sports venue, a structured laboratory dyadic task and an online small-group discussion, spanning a gradient from physically embodied group activity to text-based interaction. Each was rated by two masked observers on a common social-engagement rubric and their average served as the outcome.

(6) Subjective peer rapport

Because Hypothesis 3 predicts that objective synchrony and subjective experience respond to team composition in opposite directions, rapport was measured directly rather than inferred from the caregiver report. A five item pictorial scale was adapted from the rapport items used in cross neurotype interaction research (Crompton et al., 2020), asking about enjoyment of working with the partner, ease of coordination, comfort at close range in the water, willingness to rejoin the same team and felt understanding. Items use a seven point pictorial scale anchored by schematic faces and the score is their mean, with higher values indicating greater rapport; internal consistency was Cronbach's α = 0.86 and two week test retest reliability ICC = 0.79.

The scale was completed at T0 and T2. So that a baseline rating of the same partner exists for every participant, the T0 administration followed a standardized 20 minute familiarization session in which each participant completed one paired coordination task with the partner assigned by randomization, delivering no handoff and no training content. At T2 participants rated the teammate with whom they had most recently exchanged a handoff, or shared a matched turn window in the traditional condition. Ratings were collected by an assessor who did not observe the session and was not informed of team composition, and missing T2 values were completed within the same imputation model as the primary outcome. Item wording and administration are given in Supplementary material 4.

### 3.6 Data analysis plan

All analyses were pre-specified before data lock and followed an intention-to-treat principle. The intention to treat sample for the factorial analyses is the 192 adolescents with autism randomized to the six cells; the 32 of the WLC group enter the secondary contrasts only and the 64 peers supply no outcome data. Missing outcome values were completed by multiple imputation with chained equations (van Buuren and Groothuis-Oudshoorn, 2011) under a missing-at-random assumption, with *m* = 20 imputed sets pooled by Rubin's rules and Barnard and Rubin adjusted degrees of freedom, and complete case analysis was pre-specified as a sensitivity analysis. Baseline equivalence across the seven groups was tested with one-way ANOVA and chi-square tests.

H1 was tested with a 2 (training mode) × 3 (pairing strategy) ANCOVA on SRS-2 change from T0 to T2, with baseline SRS-2 as covariate. Analysis of covariance is the model used for every factorial test on this outcome; with six cells and one covariate the residual degrees of freedom are 185 in the intention to treat sample and 163 in the complete case analysis. The secondary contrasts against the WLC group were carried out in a one way analysis of covariance across the seven randomized groups with the same covariate, which carries 216 residual degrees of freedom on the 224 randomized participants and supplies the residual mean square entering Equation (5). Significant interactions were followed by Bonferroni-corrected simple effects, with partial eta-squared for omnibus tests and Cohen's d for pairwise contrasts, and the WLC group entered a secondary contrast against each cell, for which the effect size was defined as


$$\begin{array}{l} d=\frac{{Ȳ}_{\text{adj},g}-{Ȳ}_{\text{adj},\text{WLC}}}{\sqrt{MSE}} \end{array}$$ (5)

where the numerator is the difference between the covariate adjusted mean of cell *g* and that of the WLC group and the denominator is the residual standard deviation of the model, that is the square root of the residual mean square and not the residual mean square itself. The confidence interval of *d* came from $\text{Var}\left(d\right)=n_{g}^{-1}+n_{\text{WLC}}^{-1}+d^{2}{\left[2\left(n_{g}+n_{\text{WLC}}\right)\right]}^{-1}$, computed on the same intention to treat sample of 32 per cell, and a contrast is significant only when its Bonferroni adjusted *p* falls below 0.05 and its interval excludes zero.

H2 was tested with the Preacher-Hayes bootstrap using 5,000 resamples and bias-corrected intervals, with *W*_ARSCW_ as mediator, SRS-2 change as outcome and dummy-coded training mode and pairing strategy as predictors. The mediator is the mean over the windows of weeks 10 to 12, measured before the T2 outcome, and both arms supply observed values; baseline *W*_ARSCW_ and baseline SRS-2 entered as covariates and a random intercept for relay team was retained. A model restricted to the relay arm, with pairing strategy replacing training mode, and a complete case analysis were pre-specified as robustness checks.

H3 was tested on the rapport score with a 2 × 3 ANCOVA of the same specification as the H1 model, using baseline rapport as covariate, followed by Bonferroni corrected pairwise comparisons among the three pairing conditions, and the correlation between change in rapport and change in *W*_ARSCW_ quantified the predicted dissociation.

H4 was evaluated by regressing the composite generalization score at T3 on baseline synchrony and on intervention-induced synchrony gain, controlling for training mode. The dose response relation was expressed against the cumulative window count *n*_*w*_, the exposure variable, not against *W*_ARSCW_, the depth variable. Sensitivity analyses under moderate missing-not-at-random informativeness confirmed robustness. Analyses used R 4.3 (mediation; mice; lme4 and lmerTest with Satterthwaite degrees of freedom), alpha 0.05. Every factorial model was additionally fitted as a linear mixed model with a random intercept for relay team and a crossed random effect for peer identity, and the intraclass correlation, design effect and peer level variance component are reported beside the fixed effects.

## 4 Experimental results

### 4.1 Baseline balance and intervention fidelity

A total of 224 adolescents with autism were randomized, 192 to the six cells at 32 per cell and 32 to the WLC group; the 64 peers were allocated 32 to each arm, took part in training only and were excluded from every analysis. During the intervention 26 participants withdrew (11.6% attrition) because of family relocation (8), academic conflicts (7), parental withdrawal (6), mild adverse reactions (3) and loss of contact (2), with no serious adverse events and no significant difference in attrition across the seven groups by Fisher's exact test (*p* = 0.987). The observed sample was 198 at T2, of whom 170 were in the six cells, and 189 at T3. The intention to treat sample remains 192; the 22 missing T2 values in the cells and 4 in the WLC group were completed by multiple imputation, so every factorial statistic is reported on 192 participants with 185 residual degrees of freedom, every complete case statistic on 170 with 163, and the seven group model behind the secondary contrasts on 224 with 216.

Baseline demographic and clinical characteristics are presented in Table 3. The seven groups showed no significant differences on any baseline variable, indicating that stratified randomization achieved the expected balance.

**Table 3:** Comparison of baseline demographic and clinical characteristics across intervention cells.

Group	*n* randomized	Age (years)	Male (%)	Intelligence quotient	SRS-2 total score	ADOS-2 calibrated total score
Relay: two participants with autism	32	14.5 ± 1.6	78.1	91.8 ± 11.9	77.2 ± 8.4	7.6 ± 1.8
Relay: participants with autism and typical development	32	14.7 ± 1.8	75.0	93.1 ± 12.4	76.1 ± 9.1	7.3 ± 1.9
Relay: mixed team	32	14.4 ± 1.7	81.3	92.4 ± 11.7	76.9 ± 8.8	7.5 ± 1.7
Traditional: two participants with autism	32	14.6 ± 1.5	78.1	92.9 ± 12.1	77.5 ± 8.6	7.7 ± 1.9
Traditional: participants with autism and typical development	32	14.8 ± 1.6	71.9	91.6 ± 13.0	76.4 ± 9.3	7.4 ± 1.8
Traditional: mixed team	32	14.5 ± 1.8	75.0	93.4 ± 11.8	77.0 ± 8.7	7.5 ± 1.7
Wait-list Control	32	14.7 ± 1.7	78.1	92.1 ± 12.5	77.3 ± 8.9	7.6 ± 1.8
*F* or χ^2^	-	0.42	0.68	0.51	0.36	0.44
*p*	-	0.865	0.995	0.798	0.902	0.851

Fidelity results are presented in Table 4. All six cells reached the prespecified thresholds for attendance, script adherence, evaluator ratings and coder agreement measured by κ. Coach intervention density in the relay condition fell as designed from 4.8 per session in Stage I to 1.6 in Stage IV, matching the design curve in Figure 2, while the traditional condition remained between 3.5 and 4.2, confirming the intended contrast.

**Table 4:** Summary of intervention fidelity assessment.

Assessment Dimension	Group Relay Condition (three cells combined)	Traditional Group Swimming Condition (three cells combined)
Attendance (%)	93.6 ± 4.2	91.8 ± 4.9
Program script adherence (%)	95.1 ± 3.4	94.6 ± 3.7
Rating by independent evaluator (0 to 10 scale)	8.7 ± 0.8	8.6 ± 0.9
Coder Cohen's κ	0.86	0.84
Attrition rate (%)	12.5	10.4

### 4.2 Effects between groups and interaction between training mode and pairing strategy for core social interaction outcomes

The primary outcome differentiated clearly among groups. As shown in Figure 6, the seven groups differed across the four time points, with a steeper decline under relay than traditional swimming and only narrow fluctuation around baseline in the WLC group. Under relay with mixed teams, 62.5% of participants reached clinically significant improvement (≥ 8-point SRS-2 decrease), against 8.3% under traditional swimming with two-autism pairings.

**Figure 6:**
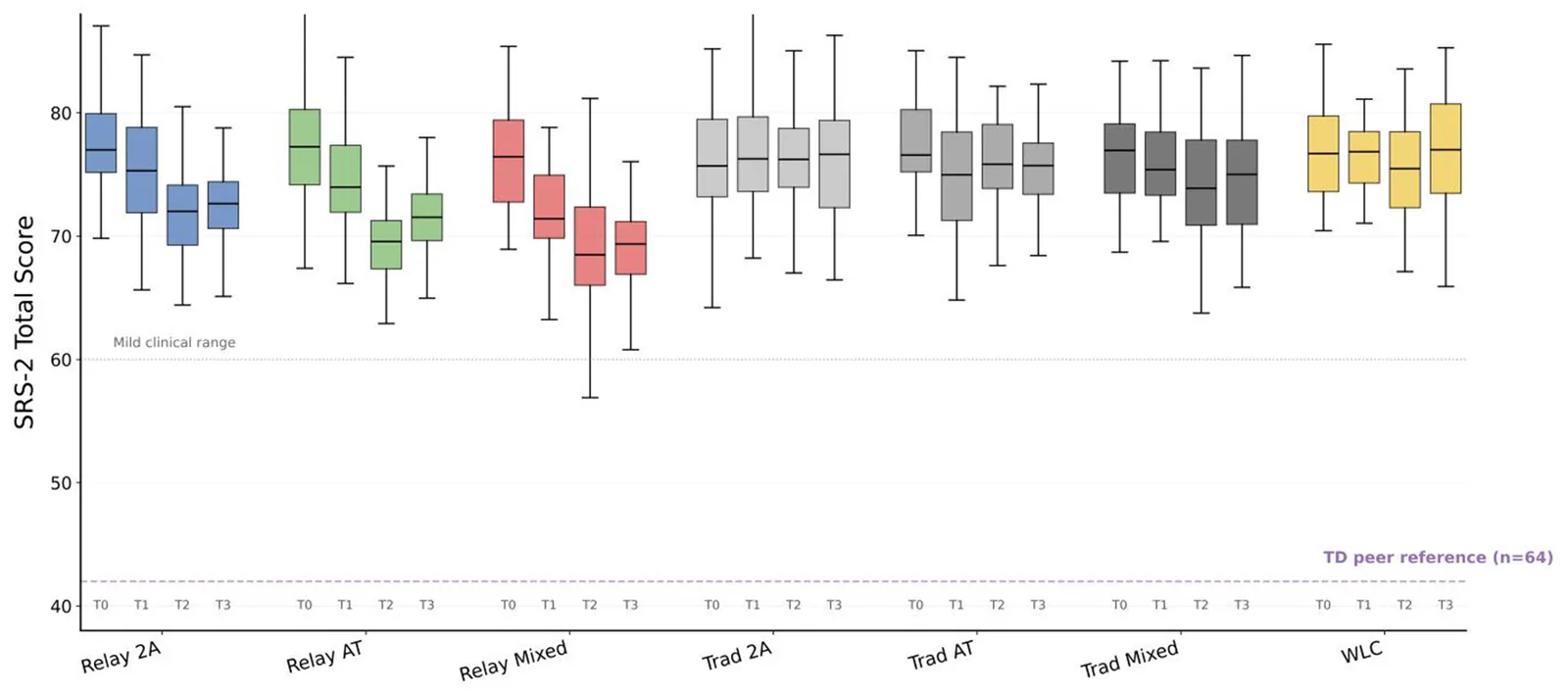
Violin plot matrix of changes in SRS-2 total scores across seven groups and four time points.

The complete results of the 2 × 3 analysis of covariance, with baseline SRS-2 as covariate, are in Table 5. Training mode showed a significant main effect, *F*_(1, 185)_ = 47.83, *p* < 0.001, partial η^2^ = 0.205, the T2 change being −7.15 ± 5.28 under relay against −2.52 ± 4.23 under traditional swimming. Across the six cells the changes were −4.6 ± 4.7, −7.6 ± 5.0 and −9.0 ± 5.3 under relay and −2.3±4.1, −2.4±4.2 and −2.7±4.4 under traditional swimming, against −0.90±3.86 in the WLC group. Pairing strategy was significant, *F*_(2, 185)_ = 5.72, *p* = 0.004, partial η^2^ = 0.058, with mixed teams and autism to typical development pairings exceeding two-autism pairings, adjusted *p* < 0.001 and *p* = 0.015, and the interaction was significant, *F*_(2, 185)_ = 4.13, *p* = 0.018, partial η^2^ = 0.043, simple effects showing differentiation among pairing strategies only under relay training, adjusted *p* > 0.10 under traditional swimming. The complete case analysis on 170 participants reproduced the pattern, *F*_(1, 163)_ = 44.62, *p* < 0.001, *F*_(2, 163)_ = 5.28, *p* = 0.006 and *F*_(2, 163)_ = 3.85, *p* = 0.024, as did the mixed model with a random intercept for relay team and a crossed random effect for peer identity, *F*_(1, 72.6)_ = 43.17, *p* < 0.001, *F*_(2, 72.6)_ = 5.11, *p* = 0.008 and *F*_(2, 72.6_) = 3.92, *p* = 0.024. The team level intraclass correlation was 0.058, giving design effects of 1.17 and 1.06, and the peer identity variance component accounted for 0.9 percent of total variance, *p* = 0.41.

**Table 5:** Results of the 2 × 3 analysis of covariance for change in SRS-2 total score.

Effect	df	*F*	*p*	Partial η^2^	Effect size interpretation
Baseline SRS-2 (covariate)	1, 185	15.36	<0.001	0.077	Medium
Training mode (relay versus traditional)	1, 185	47.83	<0.001	0.205	Large
Pairing strategy	2, 185	5.72	0.004	0.058	Small
Training mode × pairing strategy	2, 185	4.13	0.018	0.043	Small
Residual within groups	185	-	-	-	-

Each cell was then contrasted with the WLC group in the seven group analysis of covariance, using covariate adjusted means and the residual standard deviation of that model on the intention to treat sample of 32 per cell. As shown in Table 6, the three relay cells all improved significantly, with adjusted *p* of 0.009, below 0.001 and below 0.001 and intervals excluding zero, whereas no traditional cell reached significance after Bonferroni correction, with adjusted *p* of 1.000, 1.000 and 0.523 and intervals crossing zero, so the superiority of the relay format over no intervention holds for every pairing strategy.

**Table 6:** Adjusted effect sizes and bonferroni corrected comparisons of each intervention cell against the wait-list control group.

Intervention cell	Adjusted mean change in SRS-2	*d* versus WLC	95% CI	Adjusted *p*
Relay: two participants with autism	−4.57	0.83	[0.32, 1.34]	0.009
Relay: autism and typical development	−7.57	1.51	[0.96, 2.07]	<0.001
Relay: mixed team	−8.99	1.85	[1.27, 2.44]	<0.001
Traditional: two participants with autism	−2.28	0.31	[−0.18, 0.80]	1.000
Traditional: autism and typical development	−2.47	0.36	[−0.13, 0.85]	1.000
Traditional: mixed team	−2.72	0.41	[−0.09, 0.91]	0.523
Wait-list Control	−0.90	Reference	-	-

Adjusted means are least squares means and differ slightly from the observed cell means.

As shown in Figure 7, the effect size matrix and the marginal mean curves differentiate clearly. The *d* value is the adjusted cell mean minus the WLC mean divided by the residual within-groups standard deviation, that is $\sqrt{MSE}$ = 4.42, corresponding to a residual mean square of 19.54, so 4.42 is a standard deviation in SRS-2 points and not a mean square; intervals came from the large sample variance of the standardized mean difference on the intention to treat sample before Bonferroni correction. The relay mixed-team cell produced the largest effect, *d* = 1.85, 95% CI [1.27, 2.44], and the smallest was the traditional two-autism cell, *d* = 0.31, 95% CI [−0.18, 0.80], whose interval crosses zero, as do those of the other traditional cells, *d* = 0.36, 95% CI [−0.13, 0.85] and *d* = 0.41, 95% CI [−0.09, 0.91], indicating that traditional swimming alone had limited social effects. The marginal curves showed a pronounced slope for pairing strategy under relay training but were nearly flat under traditional swimming, supporting H3.

**Figure 7:**
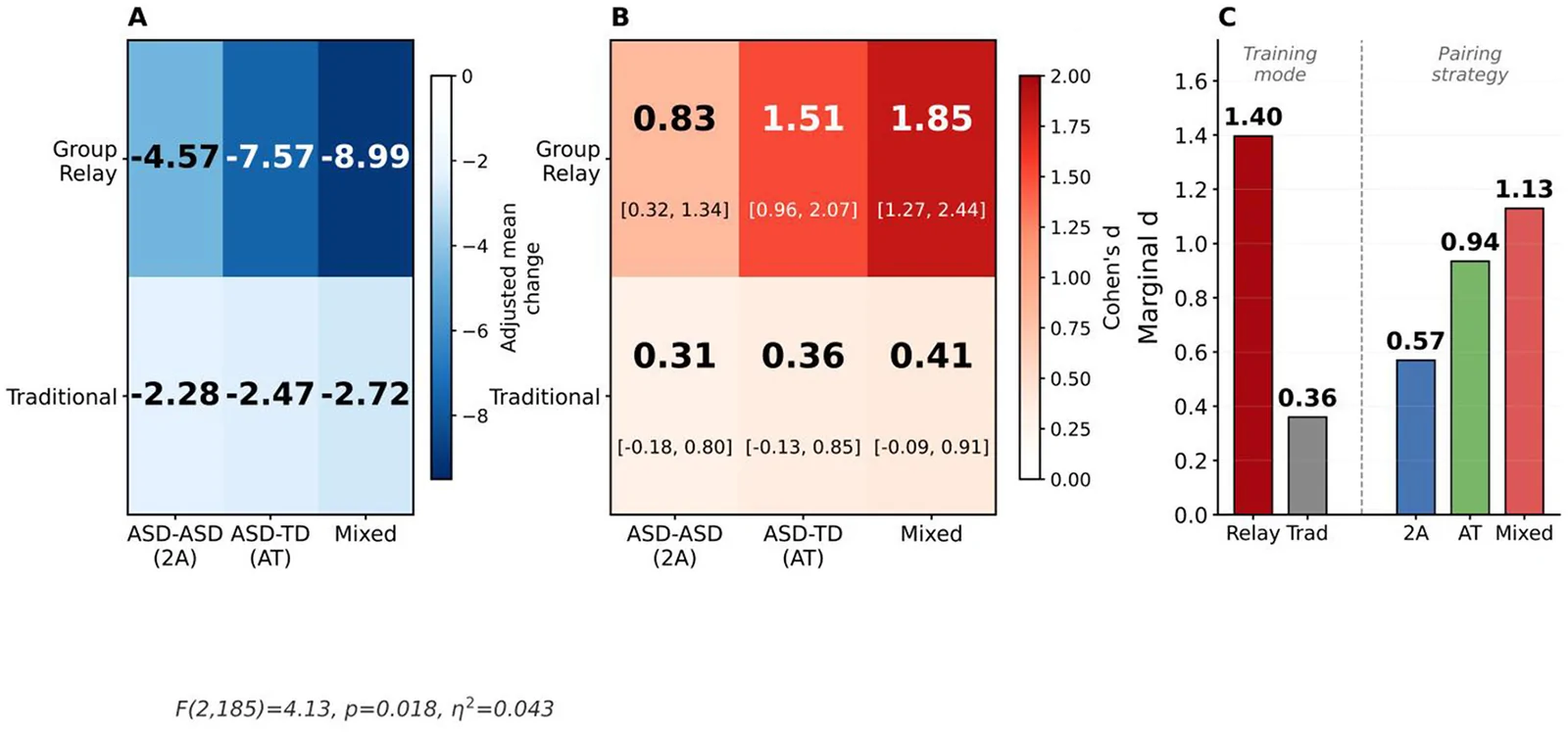
Effect size heatmap and marginal mean curves for training mode × pairing strategy. **(A)** Adjusted SRS-2 change (T2); **(B)** Cohen's d vs WLC with 95% CI; **(C)** Marginal effect sizes.

### 4.3 Dynamic Changes in Underwater Motor Synchrony, Physiological Coupling, and Behavioral Coding

The evolution of *R*(*t*) over the 12 weeks is shown in Figure 8, in which every cell of the map is the session level mean of *R*(*t*) across a whole session. At baseline the mean *R* values for the three pairing conditions ranged from 0.34 to 0.36. Under relay the curve rose first in Week 4, when dyadic coordination began, and again in Week 7, when small-group relay began, stabilizing between 0.45 and 0.56 by Week 12, whereas traditional swimming stayed within 0.04 of its starting level without systematic increase.

**Figure 8:**
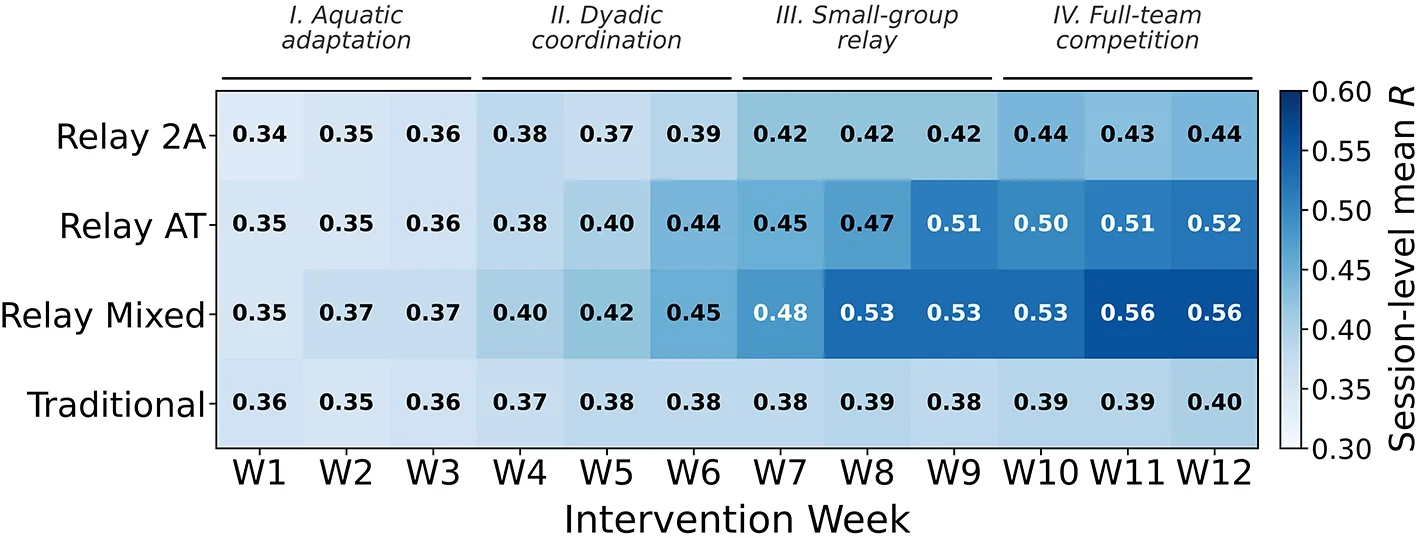
Spatiotemporal heatmap of the underwater kuramoto order parameter *R* Across the 12 weeks of training.

As shown in Table 7, the increase in session level *R* reached 0.21±0.07 for relay mixed teams against 0.11±0.05 in two-autism pairings, *p* < 0.001. The window level index *W*_ARSCW_ carried observed values in both arms, since the handoff window and the matched turn window were extracted by one pipeline. At T2 it reached 12.34 ± 2.24 s in mixed teams, 11.42 ± 2.13 s in autism to typical development pairings and 8.86±1.98 s in two-autism pairings, against 6.45 ± 1.97 s in the traditional cells combined, *F*_(3, 76.4)_ = 34.16, *p* < 0.001, while baseline values did not differ, *F*(3, 76.4)=0.41, *p* = 0.746, so the gains were 7.02 ± 1.72, 6.19 ± 1.68, 3.51 ± 1.61 and 1.13 ± 1.07 s in the same order. Because the window isolates the instant of closest alignment, the mean of *R*(*t*) inside it, equal to *W*_ARSCW_ divided by 15 s, reached 0.59 to 0.82 in the relay cells and 0.43 in the traditional cells, exceeding the session level means because they describe a different time scale of the same signal. Peak cardiac cross-correlation in mixed teams rose from 0.19 ± 0.07 to 0.41 ± 0.09 and EDA synchrony from 0.16 ± 0.06 to 0.33 ± 0.08, with both changes below 0.05 under traditional swimming. Cardiac coupling is the primary physiological index and EDA coupling secondary and exploratory.

**Table 7:** Comparison between groups of underwater synchrony, physiological coupling, and RHSC coding at T2.

Indicator	Relay: two participants with autism	Relay: participants with autism and typical development	Relay: mixed team	Traditional condition (three cells combined)	Group comparison *F* (*p*)
Increase in session level *R*	0.10 ± 0.05	0.17 ±0.06	0.21 ± 0.07	0.04 ± 0.04	46.72 (<0.001)
*W*_ARSCW_ at baseline (s)	5.35 ± 1.78	5.28 ± 1.84	5.29 ± 1.82	5.32 ± 1.80	0.41 (0.746)
*W*_ARSCW_ at T2 (s)	8.86 ± 1.98	11.42 ± 2.13	12.34 ± 2.24	6.45 ± 1.97	34.16 (<0.001)
Peak cardiac cross-correlation	0.32 ± 0.08	0.38 ± 0.09	0.41 ± 0.09	0.24 ± 0.07	27.53 (<0.001)
EDA synchrony	0.25 ± 0.08	0.30 ±0.08	0.33 ± 0.08	0.19 ± 0.06	22.87 (<0.001)
RHSC mutual gaze (events/window)	2.42 ± 0.87	3.58 ± 1.04	3.86 ± 1.08	1.14 ± 0.63	91.34 (<0.001)
RHSC nonverbal feedback (events/window)	1.58 ± 0.72	2.36 ± 0.91	2.61 ± 0.95	0.58 ± 0.42	69.42 (<0.001)

All group comparisons are *F* tests with 3 and 76.4 degrees of freedom from linear mixed models carrying a random intercept for relay team, with Satterthwaite degrees of freedom.

Figure 9 shows the 12-item RHSC profiles of the three relay cells and the combined traditional condition. The relay condition expanded across all four dimensions, most strongly in non-verbal feedback, where fist bumping, clapping and nodding rose from 0.58 events per window at baseline to 2.61 at T2 and remained at 2.35 at T3, so the effect did not depend on continued presence of the training context. Verbal exchange expanded more modestly, with the largest gain in encouragement (+1.42) rather than task information (+0.48).

**Figure 9:**
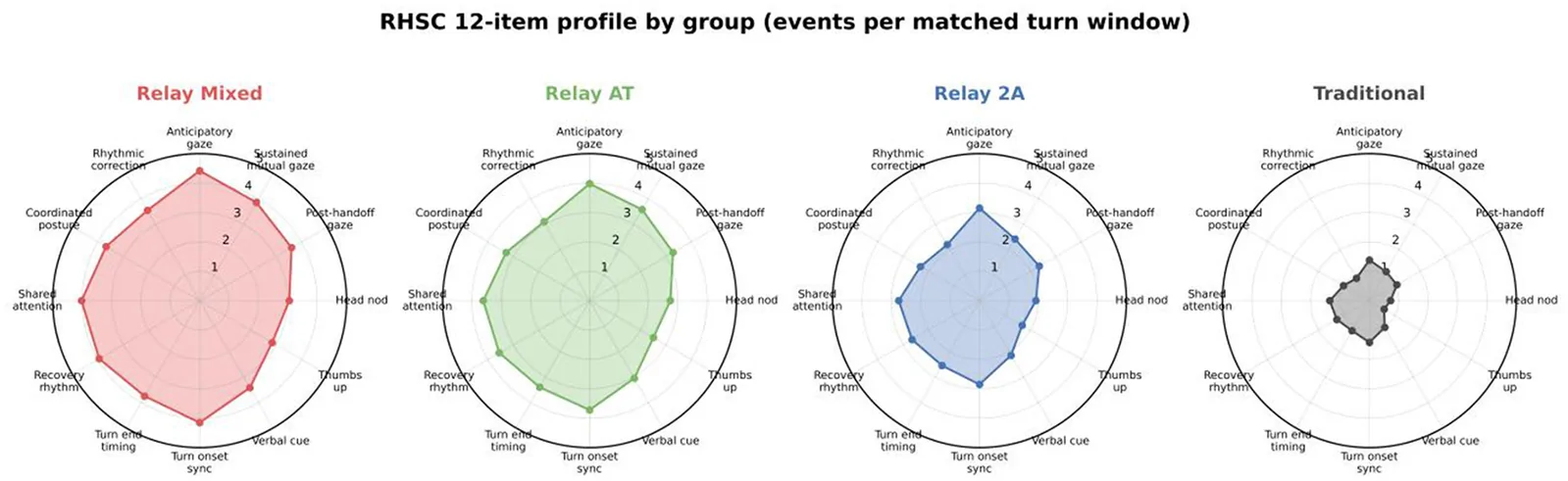
Radar chart comparison of the 12-item RHSC profiles of the three relay cells and the combined traditional condition.

### 4.4 Subjective peer rapport and its dissociation from objective synchrony

Peer rapport moved opposite to objective synchrony. At T2 the mean score was highest in two-autism pairings, 5.42 ± 0.88 under relay and 5.18 ± 0.92 under traditional swimming, lowest in autism to typical development pairings, 4.61 ± 0.96 and 4.49 ± 0.99, and intermediate in mixed teams, 4.83 ± 0.91 and 4.66 ± 0.94. The ANCOVA with baseline rapport as covariate gave a significant main effect of pairing strategy, *F*_(2, 185)_ = 13.10, *p* < 0.001, partial η^2^ = 0.124, a non-significant main effect of training mode, *F*_(1, 185)_ = 2.03, *p* = 0.156, and a non-significant interaction, *F*_(2, 185)_ = 0.08, *p* = 0.924. Bonferroni corrected comparisons placed two-autism pairings above autism to typical development pairings, adjusted *p* < 0.001, and above mixed teams, adjusted *p* = 0.001, while the latter two did not differ, adjusted *p* = 0.570 (Table 8).

**Table 8:** Subjective peer rapport at T2 by training mode and pairing strategy.

Pairing strategy	Group relay condition	Traditional group swimming condition	Marginal mean	Bonferroni corrected comparison
Two participants with autism	5.42 ± 0.88	5.18 ± 0.92	5.30	Higher than the other two conditions, adjusted *p* < 0.001 and *p* = 0.001
Participants with autism and typical development	4.61 ± 0.96	4.49 ± 0.99	4.55	Not different from the mixed team, adjusted *p* = 0.570
Mixed team	4.83 ± 0.91	4.66 ± 0.94	4.75	Not different from the cross neurotype pairing, adjusted *p* = 0.570

The two dimensions therefore separate rather than converge. The strategy producing the largest gain in *W*_ARSCW_, the mixed team, was rated 0.55 scale points below two-autism pairings, and across the intervention sample the change in rapport correlated weakly and negatively with the change in *W*_ARSCW_, *r* = −0.18, *p* = 0.012. As shown in Figure 10, the rapport distributions and the corresponding synchrony gains run in opposite order along the same axis, so H3 is supported on both components.

**Figure 10:**
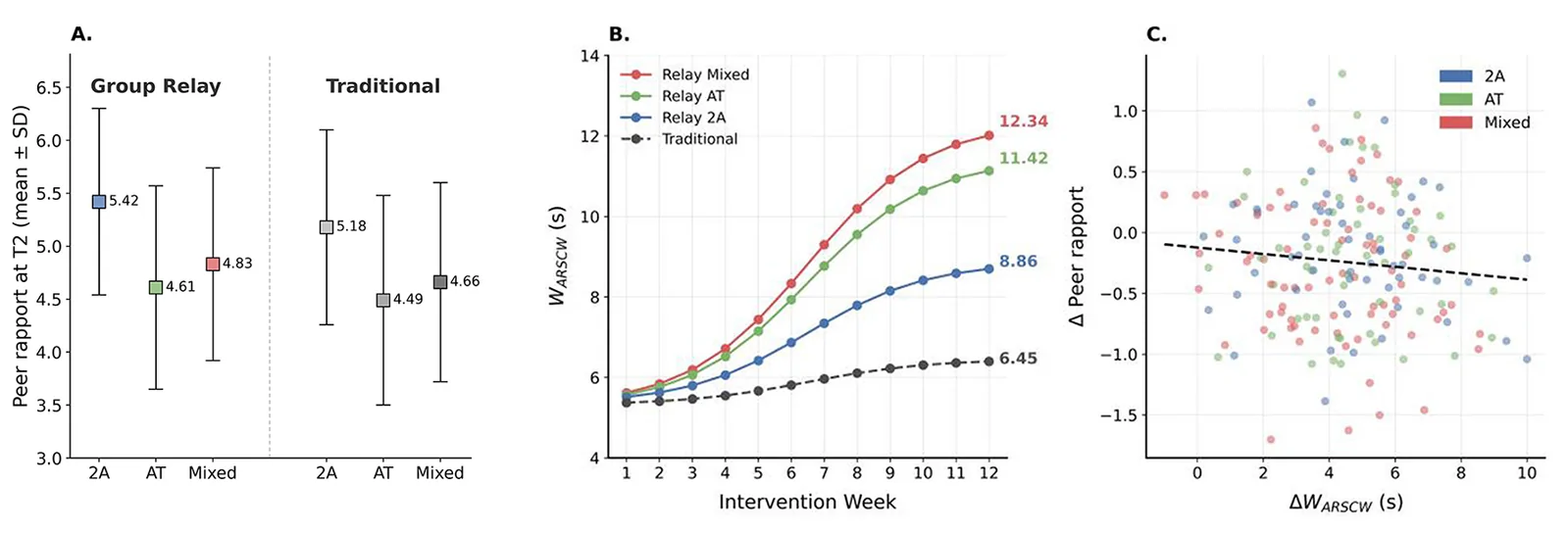
Subjective peer rapport and gains in the coupling window synchrony index across the three pairing strategies. **(A)** Peer rapport (opposite ordering); **(B)** *W*_ARSCW_ trajectories; **(C)** Dissociation, *r* = −0.18.

### 4.5 Mediation pathway of the ARSCW model and validation of generalization to contexts outside the water

The test of H2 used the Preacher-Hayes bootstrap with 5000 samples; standardized coefficients and significance levels are in Figure 11. The mediator entered as the mean over the windows of weeks 10 to 12, carried observed values in both arms, and was accompanied by baseline *W*_ARSCW_ and baseline SRS-2 as covariates and a random intercept for relay team.

**Figure 11:**
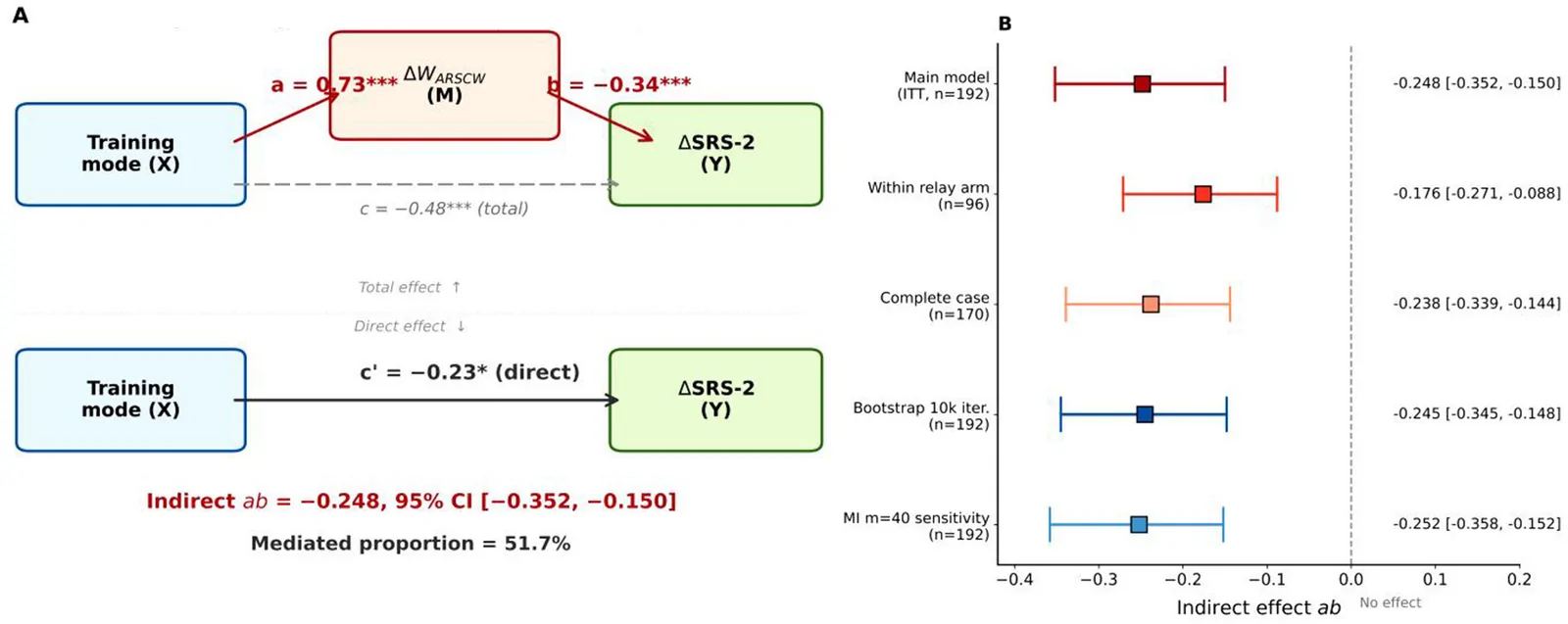
Results of the ARSCW mediation path analysis. **(A)** Mediation path diagram (main model, ITT *n* = 192); **(B)** Robustness across analytic choices.

As presented in Table 9, path *a* was 0.73, 95% CI [0.57, 0.89], *p* < 0.001, and path *b* was −0.34, 95% CI [−0.48, −0.20], *p* < 0.001. The indirect effect ab was −0.248, 95% bootstrap CI [−0.352, −0.150], excluding zero, so the IMS pathway in H2 was supported with a mediated proportion of approximately 52%; the remainder arises from competitive motivation, coach intervention and unmeasured pathways. Two pre-specified checks reproduced this: within the relay arm alone ab = −0.176, 95% CI [−0.271, −0.088], a proportion of 43.5%, and in the complete case sample ab = −0.238, 95% CI [−0.339, −0.144], a proportion of 50.6%, so the pathway does not rest on the contrast between training modes alone and survives removal of the imputed values.

**Table 9:** ARSCW mediation effect analysis based on 5000 bootstrap samples.

Path	Standardized coefficient	Standard Error	*t*	95% CI	*p*
*a* (training mode → *W*_ARSCW_)	0.73	0.08	9.13	[0.57, 0.89]	<0.001
*b* (*W*_ARSCW_ → change in SRS-2)	−0.34	0.07	−4.86	[−0.48, −0.20]	<0.001
*c* (total effect)	−0.48	0.09	−5.33	[−0.66, −0.30]	<0.001
c'	−0.23	0.09	−2.56	[−0.41, −0.05]	0.011
ab (indirect effect)	−0.248	0.052	-	[−0.352, −0.150]	-
Mediated proportion *ab*/*c*	51.7%	-	-	—	-
ab within the group relay arm	−0.176	0.046	-	[−0.271, −0.088]	-
Mediated proportion within the group relay arm	43.5%	-	-	-	-
ab in the complete case sample	−0.238	0.050	-	[−0.339, −0.144]	-
Mediated proportion in the complete case sample	50.6%	-	-	-	-

H4 was tested at T3 across five contexts. As shown in Figure 12, four of five produced effect sizes above *d* = 0.30, the largest at the community sports venue, *d* = 0.68, 95% CI [0.37, 0.99], then peer cooperation at school recess, *d* = 0.53, and the smallest in online group discussion, *d* = 0.28, 95% CI [−0.02, 0.58], crossing zero. The dose-response curve accelerated between 100 and 300 cumulative windows and approached saturation above 300, the horizontal axis being the exposure count *n*_*w*_ and not the depth index *W*_ARSCW_.

**Figure 12:**
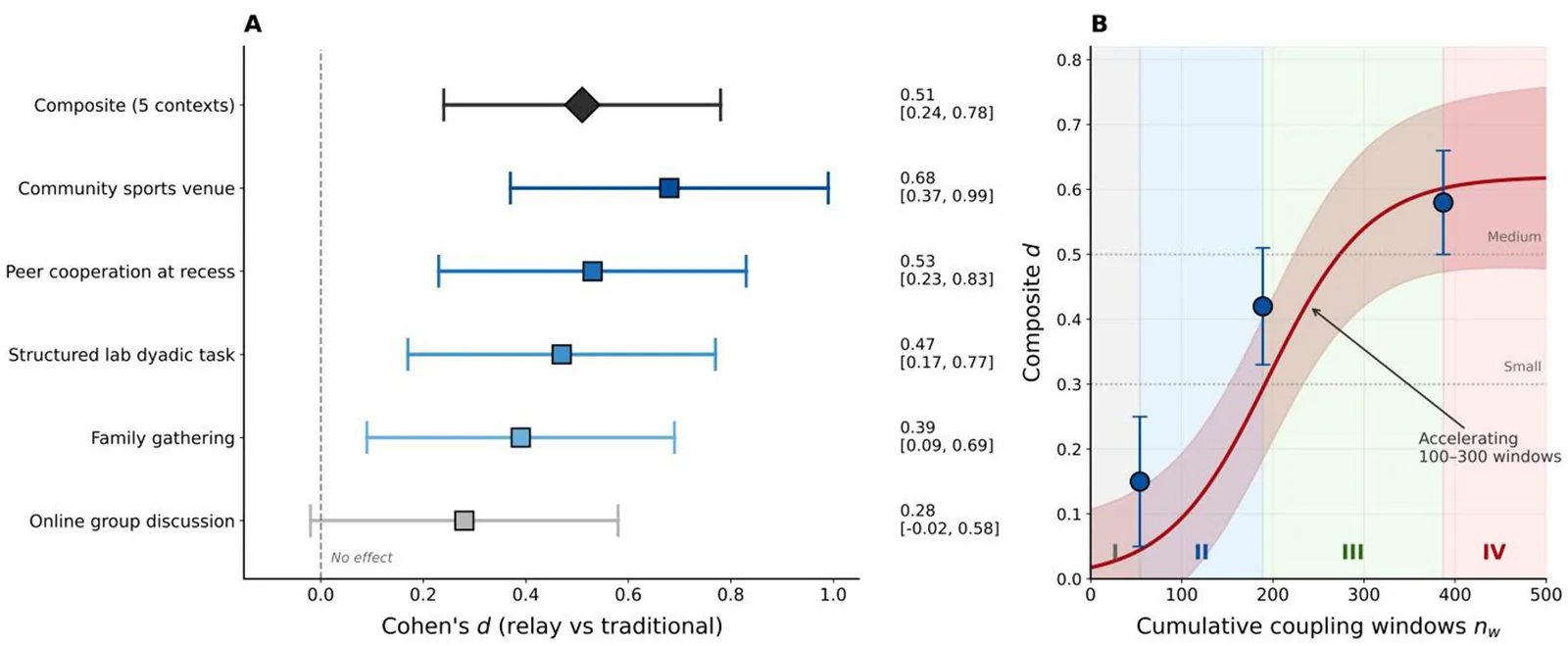
Forest plot of generalization effect sizes in nonaquatic contexts and dose-response curve. **(A)** Generalization forest plot (T3); **(B)** Dose-response curve.

As shown in Table 10, the effect sizes and the joint predictive coefficients display a clear hierarchy. Baseline IMS, β = 0.27, *p* = 0.003, and the increase in *W*_ARSCW_, β = 0.47, *p* < 0.001, independently predicted the composite generalization score, joint *R*^2^ = 0.36, supporting H4, and the predictive strength of the synchrony gain consistently exceeded that of the baseline level.

**Table 10:** Effect sizes and joint predictive coefficients for generalization to nonaquatic contexts.

Generalization Context	Effect size *d*	95% CI	Baseline IMS predictor β (*p*)	Increase in *W*_ARSCW_ predictor β (*p*)
Peer cooperation during school recess	0.53	[0.23, 0.83]	0.22 (0.018)	0.44 (<0.001)
Naturalistic observation at a family gathering	0.39	[0.09, 0.69]	0.16 (0.062)	0.34 (0.001)
Community sports venue	0.68	[0.37, 0.99]	0.31 (0.001)	0.53 (<0.001)
Structured laboratory dyadic task	0.47	[0.17, 0.77]	0.25 (0.008)	0.41 (<0.001)
Online group discussion	0.28	[−0.02, 0.58]	0.11 (0.209)	0.28 (0.006)
Composite across five contexts	0.51	[0.24, 0.78]	0.27 (0.003)	0.47 (<0.001)

Effect sizes were smaller in family gatherings and online discussions, the former reflecting the low signal to noise ratio of that context and the latter indicating that the absence of physical presence makes transfer of the core competence difficult. Taken together, H1 received support, H2 for approximately half of the total effect, H3 on both of the directions it specifies, and H4 within a boundary, since transfer was established in the four physically embodied contexts but not online, and the advantage over no intervention was confined to the three relay cells.

## 5 Discussion

### 5.1 Structural reshaping of social exposure by the relay format

The central finding was that group relay swimming improved social interaction deficits significantly more than traditional group swimming. The between-group difference in SRS-2 change reached 4.63 points and the mixed team condition produced *d* = 1.85, above the median *d* = 0.60 of previous meta-analyses of social skills interventions for autism (Afsharnejad et al., 2024), giving strong evidence for H1. The contrast against the wait list control group sharpens this: all three relay cells separated from natural change and no traditional cell did, so the advantage cannot be attributed to pool time, coach contact or group membership, all of which the active comparator matched. The most closely related recent aquatic trial points the same way, since an individually delivered aquatic program raised water competency without a reliable social gain (Kemp et al., 2024), which places the active ingredient in the structure of peer exposure rather than in immersion itself. The difference therefore reflects the structured reshaping of social exposure rather than exercise volume, the coupling window compressing each handoff into a controllable unit that permits close peer interaction under low sensory load, predictable rhythm and competitive goal orientation.

Mediation further supported H2, ab = −0.248, consistent with fNIRS evidence that creative movement activates the inferior frontal gyrus and superior temporal sulcus (Su et al., 2025). Defining the mediator as the depth of synchrony inside a fixed 15 s window extracted in both arms by one pipeline makes it a continuous property of aquatic peer coordination rather than a feature existing only where a handoff occurs, and the within arm replication, ab=-0.176, shows the pathway remains active when the relay format is held constant. Where the IMS literature treats synchrony as a phenomenological description (Carnevali et al., 2024), operationalizing it through the Kuramoto order parameter and the *W*_ARSCW_ integral turns that intuition into a reproducible quantitative conclusion.

### 5.2 Opposite effects of pairing strategy on objective synchrony and subjective rapport

The moderating effect of pairing strategy supported H3 on both directions it specifies. Objective gains were significantly greater in mixed teams and in autism to typical development pairings, with session level increases of 0.21 and 0.17 against 0.11 in two-autism pairings, whereas subjective rapport ran the other way. Reports of higher rapport between same neurotype partners (Crompton et al., 2020) are therefore not in conflict with the present results: the two literatures describe two dimensions that this design measured within one trial and found to move in opposite directions, and the weak negative correlation between their changes indicates a systematic dissociation rather than a difference between samples. Kinematic coupling depends on complementarity in motor skill, whereas felt rapport depends on shared communicative style.

The dissociation has a direct consequence for team organization. Optimizing only objective synchrony selects mixed teams and accepts the lowest rapport ratings of the three compositions, the condition under which adolescents are least willing to rejoin the same team; optimizing only rapport selects two-autism pairings and forgoes more than half of the attainable synchrony gain. Because the interaction between training mode and pairing strategy was small, partial η^2^ = 0.043, and rapport showed no interaction with training mode, the two objectives can be sequenced within one curriculum, for example by opening a block with two-autism pairings until the coupling window is reliably tolerated and shifting to mixed teams thereafter. Table 11 summarizes the correspondence between each hypothesis, its evidence and its boundary.

**Table 11:** Correspondence between hypotheses, principal evidence and boundary conditions.

Hypothesis	Principal evidence	Effect size	Boundary condition
H1 Relay training improves social interaction	Main effect of training mode; all three relay cells separate from the WLC group	Partial η^2^ = 0.205; *d* from 0.83 to 1.85	Traditional group swimming alone did not separate from natural change
H2 Synchrony mediates the effect	Indirect effect through *W*_ARSCW_, replicated within the relay arm and in the complete case sample	ab=-0.248, mediated proportion 51.7%	About half of the total effect is carried by unmeasured pathways
H3 Pairing strategy moderates the effect	Objective synchrony highest in mixed teams; rapport highest in two-autism pairings	Partial η^2^ =0.043 for the interaction on SRS-2 and 0.124 for pairing strategy on rapport	The two dimensions move in opposite directions, *r* = -0.18
H4 Transfer to contexts outside the water	Significant transfer in four of five contexts; dose response rising between 100 and 300 windows	*d* from 0.39 to 0.68 in physically embodied contexts	Online discussion, *d* = 0.28, with an interval crossing zero

### 5.3 The embodied gradient of transfer and the limits of the present evidence

H4 received differentiated support: strongest at community sports venues, *d* = 0.68, then peer cooperation at school recess, *d* = 0.53, and weakest in online discussion, *d* = 0.28, with an interval crossing zero. The gradient between physical similarity of the context and transfer magnitude indicates that the trained competence centers on temporal coordination during physical presence, so the effect declines rapidly once physical coordination is absent. This is both a boundary condition and a starting point for cross-modal transfer research.

Four limitations bound these conclusions. The electrodermal channel retained fewer usable windows and was less repeatable than the cardiac channel, so those findings are exploratory. Each peer served in two teams within one arm, a dependence the crossed random effect absorbed but did not remove, although its variance component was negligible. The wait list control group estimates natural change rather than a placebo response, so the relay advantage is established against no intervention and a matched aquatic comparator. The follow up extends to 12 weeks, enough to show that behavioral gains outlast the training context but not to establish whether the coupling window remains tolerated once the competitive structure is withdrawn.

## 6 Conclusion

This study proposed and validated the Aquatic Relay Social Coupling Window model in a 2 × 3 factorial randomized controlled trial in which 192 adolescents with autism entered six intervention cells, 32 formed a wait list control group and 64 typically developing adolescents served as training partners. The main conclusions are as follows. (1) Group relay swimming improved social interaction deficits more than traditional group swimming, with a peak adjusted effect size of *d* = 1.85 in the mixed team condition, and all three relay cells separated significantly from the wait list control group whereas no traditional cell did. (2) The depth of synchrony sustained inside the 15 s coupling window, expressed as *W*_ARSCW_ in seconds, mediated 51.7% of the total effect; the index carries observed values under both training modes and the mediation was reproduced within the relay arm and in the complete case sample. (3) Pairing strategy moderated the effect in opposite directions on the two outcome dimensions, mixed teams and autism to typical development pairings producing the larger objective gains and two-autism pairings the higher subjective rapport. (4) Acquired abilities transferred to physically embodied settings and were jointly predicted by baseline synchrony and training induced gain, whereas transfer to online discussion was not established. The model, the RHSC and the underwater synchrony pipeline form a reusable toolkit for group exercise research in autism. The sample is confined to ages 12 to 17, the follow up to 12 weeks, the electrodermal channel remains exploratory and each peer was reused across two teams; future work should widen the age range and sport modalities, lengthen the follow up and apply dyadic fNIRS to the neural coupling underlying the window.

## Data Availability

The original contributions presented in the study are included in the article/Supplementary material, further inquiries can be directed to the corresponding author.
