# Supplementary material for: An experimental study on the intervention effects of group relay swimming training on social interaction deficits in adolescents with autism
Source: Front Psychol. 2026 Oct 13;17:1976678. doi: 10.3389/fpsyg.2026.1976678 (PMC13710102; doi:10.3389/fpsyg.2026.1976678)
Supplement: Supplementary file 1 [file Supplementary_file_1.docx]

**Supplementary Material**

**S1 Stage-specific content of the group relay swimming program**

The program ran for 12 weeks with three 90-minute sessions per week. Stage I, weeks 1 to 3, was the aquatic adaptation stage. Participants practiced individual techniques side by side without physical contact in shared lanes and no relay handoff was required. Coach intervention density was highest in this stage, with frequent demonstrations, verbal cues and one-to-one adjustments, averaging 4.8 interventions per session.

Stage II, weeks 4 to 6, was the dyadic coordination stage. Pairs performed synchronized strokes guided by a waterproof metronome, followed by mirrored kicking drills and side-by-side freestyle. At the end of each set the coach displayed a rhythm-consistency score on a poolside screen as objective process feedback, shifting attention from the coach to the movement pattern shared with the partner. Each session contained approximately eight paired coordination moments structurally comparable to a handoff, providing early exposure to the coupling window, and coach intervention density averaged 3.7 per session.

Stage III, weeks 7 to 9, was the small-group relay stage. Groups of three completed 25-meter shuttle relays focused on three actions at each handoff: observation from the block, contact at the wall and controlled departure. After each handoff a 30-second verbal review was conducted in which the coach prompted each teammate to share a single observation, for example that a partner reached the wall a little earlier than before, converting the physical event into a brief peer-directed exchange without opening an unstructured social interval. Coach intervention density averaged 2.3 per session.

Stage IV, weeks 10 to 12, was the full-team competition stage. Teams of four simulated formal 4 by 50-meter freestyle relay races, with interteam competition organized as a round-robin points system. Coach intervention density was intentionally lowest in this stage, averaging 1.6 per session: the coach acted primarily as race adjudicator and safety officer, and responsibility for pacing, encouragement and post-race debriefing was transferred to the participants. This progressive withdrawal of adult scaffolding is central to the model, because durable peer coordination is expected to emerge only when adult support is removed.

Across the four stages the number of relay handoffs per session rose from 0 to 8, 18 and 24. These figures are the values reached at the end of each stage, and the number delivered in a single session rose progressively within the stage, so that the session mean of a stage is lower than its closing value. Accumulated over the nine sessions of each stage, the cumulative number of coupling windows for one participant rose from 0 to approximately 54, 189 and 387. These counts are the exposure variable used in the dose response analysis of Hypothesis 4.

**S2 Randomization: stratification and block implementation**

Allocation was generated by an independent statistician who took no part in recruitment, delivery or assessment, and was administered through a central web-based platform. The block size was fixed at 14, which equals two allocations for each of the seven groups formed by the six factorial cells and the wait list control group, so that every completed block preserved exact balance across all seven groups simultaneously.

Stratification used three variables: age band, with two levels of 12 to 14 and 15 to 17 years; sex, with two levels; and baseline SRS-2 total score, divided into three approximately equal tertiles. The three variables yield 12 strata, listed in Table S1, and an independent permuted block sequence of length 14 was generated for each stratum.

The platform enforced a recruitment cap of 32 participants for each of the seven groups and withdrew a group from all subsequent blocks once its cap was reached, so that the closing block of several strata was an incomplete block. The combination of stratum specific sequences, a block length that is an exact multiple of the number of groups, and group level caps produced the final allocation of exactly 32 participants to each of the seven groups.

**Table S1 Stratification structure and number of participants allocated per stratum**

| Stratum | Age band (years) | Sex | Baseline SRS-2 tertile | Participants allocated |
| --- | --- | --- | --- | --- |
| 1 | 12 to 14 | Male | Lower | 27 |
| 2 | 12 to 14 | Male | Middle | 25 |
| 3 | 12 to 14 | Male | Upper | 26 |
| 4 | 12 to 14 | Female | Lower | 8 |
| 5 | 12 to 14 | Female | Middle | 7 |
| 6 | 12 to 14 | Female | Upper | 8 |
| 7 | 15 to 17 | Male | Lower | 31 |
| 8 | 15 to 17 | Male | Middle | 32 |
| 9 | 15 to 17 | Male | Upper | 31 |
| 10 | 15 to 17 | Female | Lower | 9 |
| 11 | 15 to 17 | Female | Middle | 10 |
| 12 | 15 to 17 | Female | Upper | 10 |
| Total |  |  |  | 224 |

**S3 Physiological signal processing, artifact rejection and reliability**

Heart rate and heart rate variability were recorded with Polar H10 chest straps worn beneath the swimsuit, storing RR intervals on the device at 1000 Hz. Electrodermal activity and skin temperature were recorded with Empatica EmbracePlus wristbands at 4 Hz, worn inside a neoprene sleeve that limits water flow across the electrodes. Devices were fitted before each session, participants completed a five-minute quiet-standing baseline, and all streams were clock-aligned with the kinematic and video channels within 10 milliseconds.

RR series were screened with an automatic threshold detector for ectopic and missed beats set at 0.25 s, and corrected samples were replaced by cubic spline interpolation. Windows in which more than 5 percent of beats required correction were discarded. Electrodermal signals were low pass filtered at 1 Hz with a zero phase Butterworth filter and motion artifacts were labelled by a supervised detector operating on statistical and spectral features of the signal together with the triaxial acceleration recorded by the same wristband. Windows in which more than 10 percent of samples were labelled as artifact were discarded. Because a submerged electrode pair cannot separate sweat gland conductance from the conductance of the surrounding water, the electrodermal analysis was further restricted to samples in which the wrist was above the water surface, identified from the triaxial acceleration together with the wet contact flag of the device; this condition is met for most of the preparation beat and for part of the departure beat.

The cardiac quantity entering the coupling analysis was the instantaneous heart rate series obtained by resampling the corrected RR series at 4 Hz. A spectral index is not defined over a 15 s interval and was therefore not used. The root mean square of successive differences was computed over the same windows as a descriptive time domain measure. At the heart rates observed inside the coupling window, between 140 and 170 beats per minute, a 15 s segment contains 35 to 43 RR intervals, a beat count comparable to that of a 30 s segment recorded at rest.

Reliability was established in two ways. First, each retained window was compared with 1000 phase randomized surrogate pairs and the observed coupling was counted as exceeding chance when it lay above the 95th percentile of the surrogate distribution. Second, a repeated measurement substudy in which 24 participants were recorded across three consecutive sessions provided intraclass correlation coefficients of the ICC(2,1) form. The resulting statistics are listed in Table S2. Because the electrodermal channel retained fewer windows, describes only the dry phases of the window, exceeded chance less often and reached lower repeatability, cardiac coupling is treated as the primary physiological index and electrodermal coupling as a secondary exploratory index throughout the manuscript.

**Table S2 Signal quality, artifact rejection and reliability of the two physiological channels**

| Indicator | Cardiac channel | Electrodermal channel |
| --- | --- | --- |
| Sampling | RR intervals at 1000 Hz | 4 Hz |
| Quantity entering coupling analysis | Instantaneous heart rate resampled at 4 Hz | Filtered skin conductance |
| Windows retained after artifact rejection (%) | 86.2 | 79.4 |
| Windows also meeting the above water criterion (%) | Not applicable | 61.8 |
| Windows exceeding the 95th surrogate percentile (%) | 81.7 | 68.3 |
| Repeated measurement ICC(2,1), 24 participants, 3 sessions | 0.74 | 0.66 |
| Agreement of the 15 s time domain measure with a 5 min reference | r=0.84 | Not applicable |
| Status in the manuscript | Primary | Secondary and exploratory |

**S4 Peer rapport scale: items and administration**

The five item pictorial rapport scale was adapted for this study from the rapport items used in cross neurotype interaction research. Each item is answered on a seven point pictorial scale anchored by schematic faces, from 1 for the least positive response to 7 for the most positive, and the scale score is the mean of the five items, with higher values indicating greater rapport. Internal consistency in the present sample was Cronbach alpha of 0.86 and two week test retest reliability was ICC of 0.79. The item wording is given in Table S3.

**Table S3 Items of the peer rapport scale**

| Item | Wording |
| --- | --- |
| 1 | How much did you enjoy working with your partner today? |
| 2 | How easy was it to get your movements to match your partner? |
| 3 | How comfortable did you feel being close to your partner in the water? |
| 4 | How much would you like to be on the same team as this partner again? |
| 5 | How well do you think your partner understood you today? |

The scale was completed at T0 and at T2. So that a baseline rating of the same partner exists for every participant, the T0 administration followed a standardized 20 minute familiarization session in which each participant completed one paired coordination task in the water with the partner assigned by the randomization. That session delivered no relay handoff and no training content and served only to create the rated encounter. At T2 participants rated the teammate with whom they had most recently exchanged a handoff in the group relay condition, or with whom they had most recently shared a matched turn window in the traditional condition.

Ratings were collected within 10 minutes of the end of the session, in a quiet room adjacent to the pool, by an assessor who did not observe the training session and was not informed of team composition. Items were read aloud and the response recorded by the assessor. The items name only the partner and never the neurotype or the composition of the team. Rapport values missing at T2 were completed within the same multiple imputation model used for the primary outcome, so that the rapport analysis rests on the same intention to treat sample of 192 participants.
